# The Vaccine–Field Strain Gap in Bovine Neonatal Diarrhea: Molecular Epidemiology of Rotavirus, Coronavirus, and Enterotoxigenic *Escherichia coli* and Priorities for Vaccine Updating

**DOI:** 10.3390/vaccines14080702

**Published:** 2026-08-13

**Authors:** Gyeong-Seo Park, Minsung Park, Somin Lee, Chonghan Kim, Byoung Joo Seo

**Affiliations:** 1 Vaccine Lab, WOOGENE B&G Co., Ltd., Seoul 07299, Republic of Korea; gyeoungseopark@gmail.com (G.-S.P.); pms0918@woogenebng.com (M.P.); somin94@woogenebng.com (S.L.); chonghan2004@naver.com (C.K.); 2 College of Veterinary Medicine, Jeonbuk National University, Iksan 54596, Republic of Korea

**Keywords:** bovine neonatal diarrhea, bovine rotavirus, bovine coronavirus, enterotoxigenic *Escherichia coli*, maternal vaccination, molecular epidemiology, vaccine–field strain gap, antimicrobial resistance

## Abstract

Bovine neonatal diarrhea (BND) is a leading cause of morbidity and mortality in pre-weaned calves. Commercial maternal vaccines targeting bovine rotavirus (BRV), bovine coronavirus (BCoV), and enterotoxigenic *Escherichia coli* (ETEC) have been available for decades, yet field outbreaks continue in vaccinated herds. This review examined peer-reviewed literature published between January 2019 and March 2026, identified through a structured PubMed search supplemented by reference-list screening. For BRV, a field strain sharing its VP7 genotype with a vaccine component was not neutralized by vaccine-induced antiserum, indicating that genotype concordance does not predict serologic coverage. For BCoV, hemagglutinin-esterase variation and regional lineage divergence indicate surface-protein evolution, although data linking these changes to reduced efficacy are not available. For ETEC, adhesin diversity and antimicrobial resistance affect both vaccination strategy and case management. Maternal vaccination retains biological value, but field performance is constrained by colostral variability, incomplete passive transfer, and product-to-product immunogenic differences. For all three pathogens, antigenic divergence from vaccine strains is located at individual epitopes, receptor-binding domains, or adhesins rather than at the level of whole-pathogen identity. Subunit and multi-epitope antigen designs, for which proof-of-concept constructs have been reported, operate at this level, and their selection requires characterization of candidate antigens for efficacy, diversity, polymorphism, and cross-protective breadth. Such antigen improvements address the pathogen-side gap but not the passive-transfer and mucosal constraints. Functional surveillance to guide antigen updating, field trials that measure passive-transfer efficiency, and adjunctive strategies are identified as priorities for BND control.

## 1. Introduction

Neonatal calf diarrhea is the leading infectious cause of death in pre-weaned cattle worldwide. In a national longitudinal study of preweaned dairy heifer calves across 13 US states, digestive disorders were the most frequently reported clinical sign, accounting for 56.0% of all disease events, with an overall preweaning mortality rate of 5.0% [1]. Calf diarrhea imposes comparable burdens across Europe, Asia, and South America, and persists as a primary welfare and economic concern even in production systems with established preventive programs [2].

The etiology of neonatal calf diarrhea is multifactorial, with viral, bacterial, and protozoal pathogens contributing individually and through co-infection. BRV, BCoV, and ETEC are the most consistently documented pathogens across production systems and geographic regions, and they are the sole targets of commercially licensed maternal vaccines against BND in most major markets. A meta-analysis of 41 studies from 21 countries reported pooled co-infection prevalences of 2.84% for mixed BRV–BCoV, 1.64% for BRV–ETEC, and 6.69% for BRV–*Cryptosporidium* co-infections [3]. Other pathogens, including *Cryptosporidium parvum*, *Salmonella enterica*, and *Clostridium perfringens*, contribute to enteric disease in neonatal calves but are not targeted by commercially licensed vaccines in most jurisdictions and therefore fall outside the translational scope of this review. Treating BRV, BCoV, and ETEC together is deliberate rather than incidental to the review’s scope. These three pathogens are not independent subjects that happen to share a clinical syndrome; they are the combined antigenic content of the same commercial multivalent maternal vaccines, and protection against all three is delivered to the calf through a single shared mechanism, the colostral passive-transfer cascade. A vaccine updating decision therefore cannot be made for one pathogen in isolation, because the same product, the same immunization schedule, and the same passive-transfer constraints apply to all three simultaneously. Analyzing them within one framework reflects the structure of the real-world decision the review addresses; treating them separately would obscure the shared delivery mechanism and the combined nature of the products actually in use.

Commercial maternal vaccination against BRV, BCoV, and ETEC has been practiced for decades. The licensed products in most major markets share a common design: they are inactivated (killed) multivalent formulations administered to the dam in late gestation, and their antigenic content is narrow and largely conserved across manufacturers. For BRV, the antigen is centered on serotype G6, with some products adding G10; for example, one widely used trivalent vaccine contains a G6P[5] rotavirus strain, and another contains a G6P[1] strain [4]. The BCoV component is a single inactivated whole-virus antigen derived from historical reference strains such as Mebus. The ETEC component is limited to the F5 (K99) fimbrial adhesin, with F41 included in some products. This composition has remained essentially fixed since the products were first registered, which establishes the reference point against which contemporary field-strain diversity is evaluated throughout this review: the relevant question is whether a G6-centered, F5-centered, historically fixed antigen set still matches the pathogen populations now circulating in the field. Disease outbreaks continue to occur in vaccinated herds, and field surveillance data indicate that BRV detection rates do not differ substantially between vaccinated and unvaccinated calf populations [5]. The central question is not whether these vaccines have immunological value, but whether current antigen compositions and deployment strategies remain aligned with the pathogen populations circulating in contemporary field conditions. Three explanations for continued disease in vaccinated herds recur across the published literature: antigenic divergence between vaccine strains and circulating field isolates, incomplete or inconsistent passive transfer of colostral antibodies to the neonate, and limited induction of durable intestinal mucosal immunity by parenteral vaccination programs [6,7,8]. The treatment context has also shifted. ETEC-associated diarrhea must now be evaluated within a framework of expanding antimicrobial resistance (AMR), which has narrowed empirical treatment options and prompted growing interest in probiotic supplementation, microbiome-targeted metabolites, and other non-antibiotic strategies compatible with stewardship principles [9,10,11]. The convergence of vaccine limitation and AMR pressure makes integrated prevention strategies more urgent than at any previous point in the management of BND.

Throughout this review, vaccine efficacy refers to the reduction in clinical diarrhea incidence and severity in neonatal calves born to vaccinated dams under natural field exposure, not to sterile immunity, prevention of pathogen shedding, or elimination of production losses such as reduced average daily weight gain. None of the currently licensed maternal vaccines against BRV, BCoV, or ETEC is registered based on these broader endpoints, and the published field-efficacy literature is consistent with partial rather than complete protection in vaccinated herds [6].

Two related questions warrant explicit acknowledgment. First, no threshold of antigenic heterology has been established that formally triggers a vaccine updating requirement for BRV, BCoV, or ETEC in any major regulatory jurisdiction. This review does not propose a specific threshold. Instead, it argues that cross-neutralization failure against a circulating field strain, as documented for a G6P[5] isolate that shared its VP7 genotype designation with a vaccine component yet escaped neutralization by vaccine-induced antiserum [12], constitutes a more relevant criterion than nucleotide sequence identity because it directly reflects the functional consequence for antibody-mediated protection. A sequence divergence threshold that has not been validated against neutralization outcomes cannot serve as a reliable trigger for updating decisions. Second, the geographic resolution at which surveillance should operate is a practical question. Farm-level genotypic and serologic characterization of field isolates is not economically or logistically feasible as a routine standard. Regional sentinel programs, in which structured sample submission from diagnostic laboratories feeds into centralized cross-neutralization testing on a defined periodic basis, represent the minimum infrastructure capable of generating data actionable for antigen composition review. The precedent for this model exists in seasonal influenza vaccine strain selection, where the World Health Organization recommends vaccine strains based on antigenic characterization using hemagglutination inhibition assays and molecular surveillance data aggregated across regional laboratory networks, without requiring farm-level or individual-level analysis [13].

Several components of this problem have been examined in the literature, but not within a common analytical framework. A scoping review of maternal vaccination programs for neonatal calf diarrhea found the published field-efficacy evidence to be heterogeneous and often limited by study design or geographic scope, without evaluating molecular epidemiological data bearing on antigen coverage [6]. Reviews of BCoV addressed pathogen evolution and lineage diversification without linking those findings to vaccine antigen performance or to the passive-transfer cascade that determines colostral antibody availability at the calf intestinal mucosa [7,8]. No published review has integrated molecular surveillance evidence for all three vaccine-target pathogens with functional cross-neutralization data, passive-transfer variability, and the performance of emerging adjunctive platforms within a single framework oriented toward vaccine updating decisions. The practical consequence of this gap is that vaccine updating decisions for BRV, BCoV, and ETEC are currently made without a common evidentiary standard: BRV is evaluated against cross-neutralization data that exist but are not systematically applied, BCoV is evaluated against phylogenetic data without functional bridging studies, and ETEC is evaluated against adhesin coverage assumptions that have not been re-examined since original product registration. This review proposes that all three require the same standard: functional evidence of coverage against contemporary field populations. The period from 2019 to 2026 is particularly consequential: it spans the first experimental demonstration that BRV genotype concordance with a vaccine component does not guarantee neutralization [12]; the first characterization of HE receptor-binding domain insertion variants across three continents [14,15,16]; and the first randomized field trial demonstrating that postnatal oral immunoglobulin supplementation can halve diarrhea incidence in vaccinated herds [17]. These findings have not previously been evaluated within a common analytical framework, and their cumulative implications for vaccine updating decisions have not been articulated.

**Figure 1:**
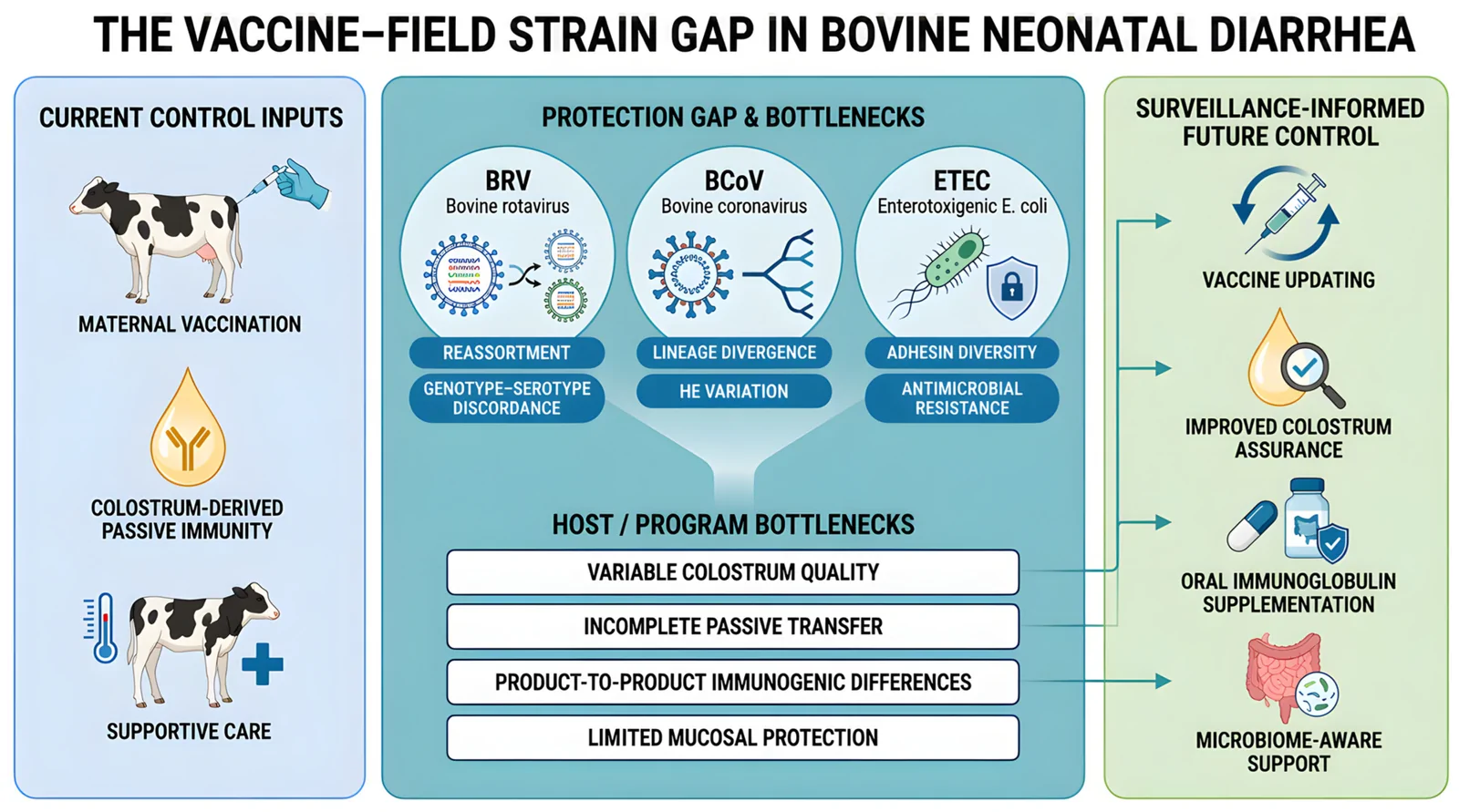
Conceptual overview of the vaccine–field strain gap in bovine neonatal diarrhea. The (**left**) panel summarizes current preventive inputs, including maternal vaccination, colostrum-derived passive immunity, and supportive care. The (**central**) panel illustrates pathogen-side mechanisms generating divergence from vaccine strains: reassortment and genotype-serotype discordance for BRV, lineage divergence and HE variation for BCoV, and adhesin diversity combined with antimicrobial resistance for ETEC, together with host- and program-level bottlenecks that limit the consistency of passive protection regardless of vaccine antigen composition. The (**right**) panel identifies surveillance-informed strategies that address these gaps: updated antigen selection, improved colostrum assurance programs, standardized oral immunoglobulin supplementation, and microbiome-aware adjunctive support.

The literature search strategy, eligibility criteria, and study selection process are detailed in Section 2 (Materials and Methods). This review examines molecular epidemiological evidence for each vaccine-target pathogen and evaluates how that evidence bears on the adequacy of current antigen compositions, the structural constraints of passive-transfer-dependent protection, and the potential of emerging platforms and adjunctive strategies to address documented gaps. A central argument is that persistent field disease in vaccinated herds reflects not a single failure point but a convergence of three independently variable factors: pathogen-side antigenic evolution, passive-transfer-cascade variability, and the absence of functional surveillance data capable of guiding antigen updating decisions. A conceptual overview is presented in Figure 1.

## 2. Materials and Methods

### 2.1. Review Design

**Figure 2:**
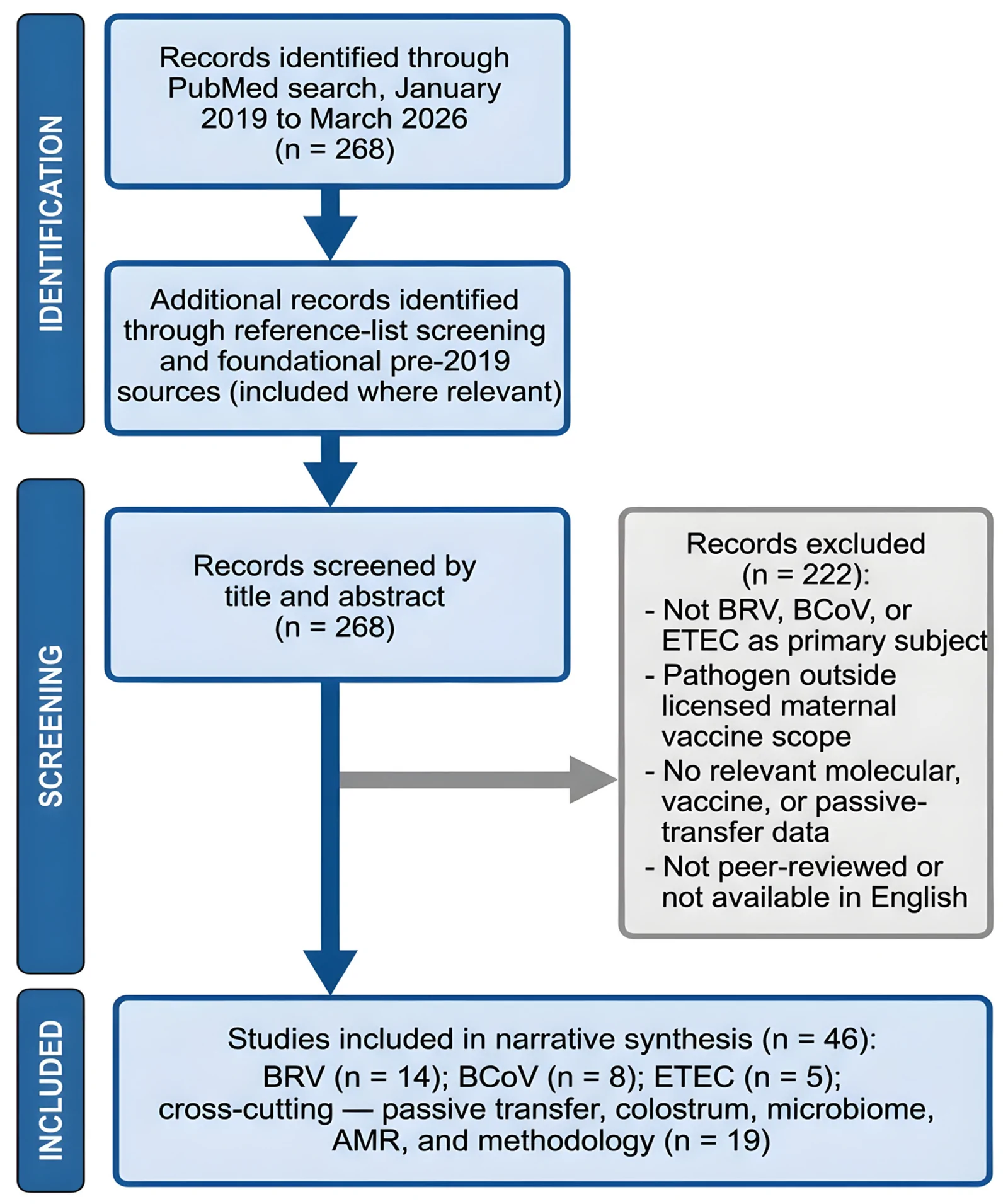
Study selection flow for the structured narrative review. A structured search of PubMed for the period January 2019 to March 2026 identified 268 records. After title and abstract screening against the predefined eligibility criteria, and with additional relevant sources identified through reference-list screening and a small number of foundational pre-2019 references, 46 studies were included in the narrative synthesis: 14 addressing bovine rotavirus (BRV), 8 addressing bovine coronavirus (BCoV), 5 addressing enterotoxigenic *Escherichia coli* (ETEC), and 19 cross-cutting studies addressing passive transfer, colostrum management, the intestinal microbiome, antimicrobial resistance, or review methodology.

This work was conducted as a structured narrative review. This format was selected because the objective was to integrate evidence across three pathogen systems (BRV, BCoV, and ETEC) and three problem domains (pathogen-side antigenic divergence, passive-transfer variability, and surveillance gaps) into a single analytical framework oriented toward vaccine antigen updating. A systematic review format was considered but not adopted. The research question is integrative and spans multiple domains rather than posing a single clinical question with a uniform outcome measure, and the evidence base does not support pooled quantitative synthesis. To support transparency and reproducibility, predefined search strategies, explicit inclusion and exclusion criteria, and a documented screening process were applied, and the study selection flow is reported in Figure 2 and Supplementary Materials File S1. The review was not registered, as prospective registration is not an established requirement for narrative reviews.

### 2.2. Search Strategy

Structured searches were conducted primarily in PubMed (MEDLINE), with Google Scholar used as a supplementary source to identify records not indexed in PubMed and to screen reference lists of retrieved articles. The primary search window was January 2019 to March 2026. The core search string applied to PubMed was: (“bovine rotavirus” OR “bovine coronavirus” OR “enterotoxigenic *Escherichia coli*” OR “neonatal calf diarrhea” OR “neonatal calf diarrhoea”) AND (“molecular epidemiology” OR “genotype” OR “antigenic” OR “cross-neutralization” OR “vaccine efficacy” OR “vaccine strain” OR “colostral immunity” OR “passive transfer” OR “antimicrobial resistance” OR “gut microbiome”). This search returned 268 records within the specified date range. Reference lists of retrieved articles were screened manually to identify additional relevant sources, and a small number of foundational references published before 2019 were included where they provided mechanistic or epidemiological context not available in more recent literature.

### 2.3. Eligibility Criteria

Inclusion was restricted to peer-reviewed primary research articles and review articles reporting: (i) pathogen-specific molecular epidemiological data for BRV, BCoV, or ETEC in cattle; (ii) vaccine immunogenicity or efficacy outcomes; (iii) passive-transfer parameters or colostral antibody data; or (iv) adjunctive intervention results relevant to bovine neonatal diarrhea. Publications were excluded if they: (i) did not address BRV, BCoV, or ETEC as the primary subject; (ii) concerned pathogens outside the scope of commercially licensed maternal vaccines, such as *Cryptosporidium*, *Salmonella*, or *Clostridium*, as the primary focus; (iii) were conference abstracts, editorials, or non-peer-reviewed preprints; or (iv) were not available in English. References published before 2019 were retained only where they provided foundational mechanistic or epidemiological context not available in more recent literature, which applied to a small number of sources on rotavirus serotype-genotype relationships and enteric pathophysiology.

### 2.4. Screening and Synthesis

Records identified through database searches were screened by title and abstract against the eligibility criteria, followed by full-text assessment of potentially relevant articles. Screening was performed by the lead author and verified by a co-author, with disagreements resolved by discussion. Because this is a narrative synthesis rather than a systematic review, formal risk-of-bias scoring of individual studies was not applied. Instead, the strength of evidence underlying each key claim is indicated in the text and classified by evidence level in Table 1, for example as a randomized controlled field trial, experimental challenge model, observational field study, or preclinical study. Included sources were organized into three analytical categories matching the review’s framework: pathogen-side antigenic divergence, passive-transfer and program-level constraints, and emerging platforms and adjunctive strategies. A structured summary of all included primary studies, listing pathogen, study design, geographic region, and key findings, is provided in Supplementary Table S1.

### 2.5. Figure Preparation

All figures in this review were rendered using FigureLabs, a figure-generation platform, from detailed text specifications and numerical data prepared by the authors. The tool was used only to render the visual output; it did not generate, calculate, estimate, or interpret any data, and it did not contribute any scientific or conceptual content. Every value, label, category, and relationship shown in the figures was specified by the authors and taken either from the primary sources cited in the corresponding figure legends or from the analytical framework developed in this review. The complete text specifications provided for each figure are reproduced in Supplementary Material File S2. No AI tool was used to generate or interpret data, to produce experimental or photographic images (none of which appear in this review), or to write the scientific content of the manuscript.

## 3. Molecular Epidemiology of BRV, BCoV, and ETEC: Evidence Relevant to Vaccine Antigen Coverage

### 3.1. Bovine Rotavirus: Genotypic Diversity, Reassortment, and Antigenic Divergence from Vaccine Strains

BRV belongs to the species Rotavirus A within the family *Sedoreoviridae* and carries an 11-segment double-stranded RNA genome. The segmented genome confers a structural predisposition to reassortment. When two or more rotavirus strains co-infect a single host cell, progeny virions may incorporate gene segments from either parent, generating novel genotype combinations within a single replication cycle. Because virus-neutralizing antibodies are directed primarily against the outer-capsid proteins VP7 and VP4, which define G and P genotypes, respectively, reassortment events involving these segments are directly relevant to the breadth of vaccine-induced immunity [23,24].

Surveillance data published between 2019 and 2026 indicate that BRV field populations across multiple continents are undergoing active genetic diversification. In the Republic of Korea, co-circulation of G6, G8, and G10 VP7 genotypes alongside P[1], P[5], and P[11] VP4 genotypes was documented in diarrheic calves, with phylogenetic evidence of multiple independent reassortment events [5]. BRV detection rates did not differ between calves from vaccinated herds (42.1%) and unvaccinated herds (42.6%), a finding more consistent with insufficient antigenic coverage than with failure of vaccine administration [4]. Cross-sectional genotyping of BRV strains isolated from regularly vaccinated dairy herds in Brazil found that circulating field strains were predominantly G6P[11] and G10P[11], genotype combinations not represented in the G6P[5] vaccine used in those herds [25].

In a vaccinated Japanese dairy operation monitored from 2018 to 2020, G6, G8, and G10 VP7 genotypes alongside P[1], P[5], and P[11] VP4 genotypes continued to co-circulate despite vaccination, with genotype composition of detected strains differing from the vaccine formulation in use [26]. These observations from geographically distinct populations reinforce the conclusion that field strain genotype composition in vaccinated herds does not reliably reflect the vaccine antigen composition. In China, whole-genome characterization identified a G6P[1] strain in which multiple gene segments were phylogenetically related to human and feline rotaviruses, providing direct evidence of interspecies reassortment extending beyond the bovine host [27]. In South America, G6 lineages showed continued spatiotemporal diversification and a G8P[11] strain was characterized within this evolutionary context [28]. Interspecies gene exchange has also been documented in a bovine G37P[52] strain genetically related to human rotaviruses isolated in the United States [29] and in a dominant Bangladeshi G6P[11] lineage in which the NSP4 gene carried intragenic recombination with human rotavirus sequences [30].

The possibility that vaccination itself may exert selective pressure on circulating genotypes adds a further dimension to this problem. In a longitudinal study of a dairy herd conducted before and after the introduction of a G6P[5] rotavirus vaccine, the dominant field strain shifted from G6-type to G10P[11] following vaccine introduction, a finding consistent with immune-mediated selection of non-vaccine genotypes under vaccination pressure [31]. This observation does not establish a causal relationship between vaccination and genotype emergence, but it suggests that long-term genotypic composition of field populations may not be stable under sustained vaccine use, further complicating the assumptions underlying fixed antigen composition strategies.

**Figure 3:**
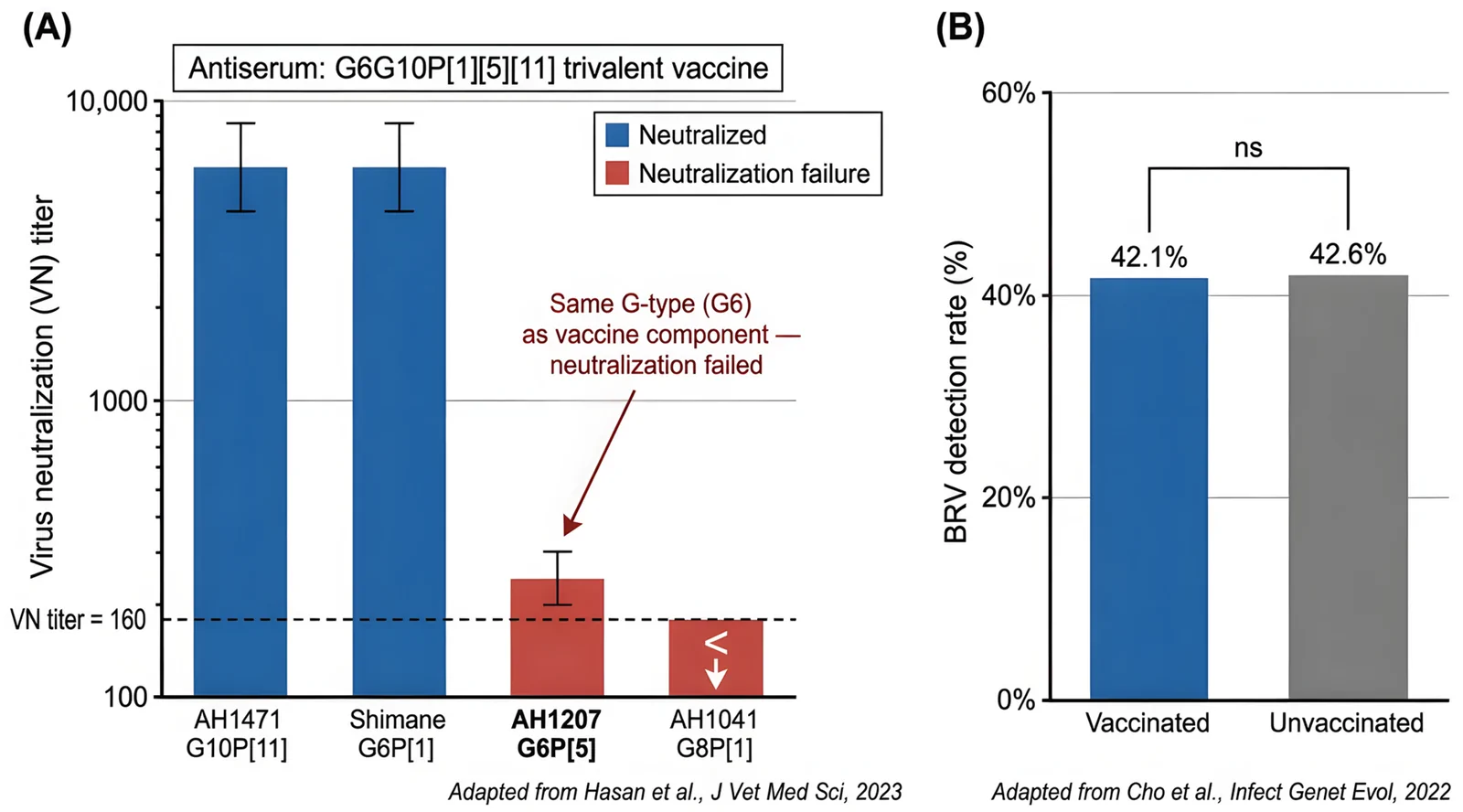
Genotype concordance does not predict serologic coverage against BRV field strains. (**A**) Virus neutralization (VN) titers of antiserum raised against a licensed trivalent BRV vaccine (G6G10P[1][5][11]) tested against four contemporary field strains. Strains AH1471 (G10P[11]) and Shimane (G6P[1]) were neutralized at high titer (3620–8791). Strain AH1207 (G6P[5]), despite sharing its VP7 genotype designation with the G6 vaccine component, showed marginal neutralization (VN titer 160–199). Strain AH1041 (G8P[1]) was not neutralized (VN titer < 160). Error bars indicate the reported titer range. The dashed line indicates VN titer = 160. (**B**) BRV detection rates in vaccinated (42.1%) and unvaccinated (42.6%) calf populations in the Republic of Korea were comparable between groups, consistent with insufficient antigenic coverage under field conditions rather than failure of vaccine administration. Data in (**A**) adapted from Hasan et al. (2023) [12]; data in (**B**) adapted from Cho et al. (2022) [5]; ns, not significant.

Genotype designation alone does not reliably predict the serologic cross-reactivity of vaccine-induced antibodies against field strains. The structural basis for this discordance has long been recognized: VP7 sequence variation can alter the conformation of neutralization epitopes without changing the broadly classified genotype designation, meaning that genotypically identical strains can differ antigenically in ways that are immunologically consequential [32]. Cross-neutralization testing of antiserum raised against a licensed Japanese trivalent BRV vaccine (G6G10P[1][5][11]) demonstrated this directly [12]. Neutralization titers against the G10P[11] field strain AH1471 and the G6P[1] strain Shimane ranged from 3620 to 8791, indicating strong homologous and cross-reactive responses. Against the G8P[1] strain AH1041 and the G6P[5] strain AH1207, neutralization was absent or marginal, with titers below 160 and between 160 and 199, respectively. The failure against G6P[5] is directly relevant to vaccine strategy: this strain shares its VP7 genotype designation with one of the vaccine components yet escaped neutralization entirely. Genotype concordance between vaccine and field strain, therefore, does not guarantee serologic coverage. RT-PCR genotyping captures sequence variation but provides no information on whether that variation alters antibody binding at neutralization epitopes. The implication is structural: a surveillance system that terminates at genotype classification will systematically fail to detect the antigenic divergence that determines vaccine coverage. For BRV, the transition from genotype-based to serology-based surveillance criteria is not a refinement of current practice but a prerequisite for any evidence-based antigen updating process. Cross-neutralization titer profiles and field detection rate comparisons illustrating this discordance are presented in Figure 3.

### 3.2. Bovine Coronavirus: Lineage Divergence and Hemagglutinin-Esterase Variation

BCoV is an enveloped, positive-sense single-stranded RNA betacoronavirus associated with three clinical presentations in cattle: enteric disease in neonatal calves, winter dysentery in adult animals, and respiratory disease in animals of various ages. Whether enteric and respiratory BCoV strains represent genetically distinct subpopulations or a shared gene pool with variable tissue tropism remains unresolved; current molecular evidence supports partial genetic overlap between the two [7,8].

Phylogenetic classification broadly divides globally circulating BCoV strains into European and Asian-American lineages, with subsequent regional diversification within each group. Korean BCoV strains isolated from diarrheic pre-weaned native calves between 2019 and 2021 clustered within the Asia/USA lineage but showed an independently evolving trajectory with comparatively elevated nucleotide substitution rates across multiple genomic regions [33]. Licensed vaccines were formulated against historical reference strains [7,8], and the degree of genetic divergence between vaccine and field strains varies by region. In Thrace, Türkiye, circulating BCoV strains showed 96.8–100% nucleotide sequence similarity to the licensed modified-live vaccine reference strain, whereas strains from geographically distant regions, including the Republic of Korea (96.6–97.8%) and the United States (96.0–97.2%), showed comparatively lower similarity to that same reference [34]. These regional differences in vaccine-field strain genetic distance indicate that the extent of antigenic divergence, and therefore its potential functional consequences, is not uniform across markets and underscores the need for region-specific assessments rather than globally generalized conclusions. A specific caution applies to the interpretation of these nucleotide identity values. A similarity of 96 to 100% may appear to indicate close antigenic correspondence, but sequence identity alone does not reliably predict antigenic relatedness in BCoV, and no fixed threshold of nucleotide divergence has been established at which neutralization escape becomes likely. The whole-genome study that characterized the U.S. HE receptor-binding domain variants concluded that whether the observed structural changes alter antibody recognition and produce immune-escape phenotypes remains to be determined, and that no consistent genetic or antigenic markers have yet been defined for BCoV [14]. Direct antigenic measurement, rather than sequence comparison, is therefore required. A recent characterization of western European BCoV isolates using virus neutralization assays demonstrated this directly: despite high genomic conservation, with spike amino-acid identities of approximately 96% relative to classical strains, antiserum raised against the Mebus vaccine strain neutralized contemporary field isolates at titers reduced 4- to 16-fold relative to the homologous strain, whereas antiserum raised against a recent isolate neutralized both recent and Mebus strains with less than a 4-fold reduction [35]. In the same work, immune escape was operationally defined as a reduction in neutralization titer of 8-fold or greater. These findings establish that a high percentage nucleotide identity can coexist with functionally significant antigenic divergence, and they identify functional neutralization testing, not sequence comparison, as the appropriate basis for judging whether BCoV field strains have drifted from vaccine antigens.

Structural variation in the hemagglutinin-esterase (HE) receptor-binding domain has been identified across multiple regions. A BCoV variant carrying a 12-nucleotide insertion in the HE receptor-binding domain was first identified in China [14]. Related insertion and deletion variants in the same domain were subsequently detected in the United States [15], and in Chinese dairy-calf populations [16]. Molecular docking analyses in the latter study suggested that the insertions alter binding affinity for O-acetylated sialic acid, the receptor recognized by HE, with possible effects on attachment efficiency. These structural observations are biologically relevant. Current evidence establishes that the field population has diverged genetically from historical vaccine strains in functionally important surface proteins. Whether this divergence translates into reduced neutralization susceptibility or diminished protection under field conditions has not been tested: no published challenge or serum neutralization study has directly compared vaccine efficacy against HE-variant or lineage-divergent strains and vaccine reference strains under controlled conditions.

This absence of functional bridging data is the critical unresolved question for BCoV vaccine assessment. Without it, the field cannot distinguish between three interpretations of continued BCoV disease in vaccinated herds: inadequate antigen coverage from strain divergence, insufficient colostral antibody transfer, or sub-protective luminal antibody concentrations at the intestinal challenge site. Each interpretation leads to a different corrective action: antigen updating, improved colostrum management protocols, or mucosal delivery alternatives, respectively, and the current evidence base cannot select among them. The experimental design that would resolve this ambiguity is not complex: a controlled challenge study comparing clinical outcomes and viral shedding in calves from dams vaccinated with current licensed products against both reference strains and contemporary HE-variant isolates, with colostral IgG concentrations and intestinal IgA titers measured as covariates. That such a study has not been conducted despite decades of BCoV vaccination practice represents the core evidentiary gap this section identifies. The precedent set by SARS-CoV-2 research is instructive in this respect: the functional consequence of spike protein and receptor-binding domain variants for vaccine-induced neutralization was established through controlled serum neutralization assays against variant isolates conducted within months of variant emergence. No equivalent methodology has been applied to BCoV field variants despite structurally analogous genetic changes in the HE receptor-binding domain.

### 3.3. Enterotoxigenic Escherichia coli: Adhesin Repertoire, Co-Infection Patterns, and Antimicrobial Resistance

ETEC causes diarrhea in neonatal calves primarily during the first four days of life through sequential colonization of the small intestinal mucosa via fimbrial adhesins and elaboration of heat-stable (ST) and/or heat-labile (LT) enterotoxins that disrupt intestinal fluid and electrolyte transport. The fimbrial adhesins F5 (K99) and F41 are the principal antigens targeted by currently licensed maternal vaccines, but the adhesin repertoire documented in field isolates of ETEC from diarrheic calves extends beyond these two antigens. A systematic review and meta-analysis of 106 publications covering 25,982 *E. coli* isolates from 27 countries established that F17 fimbriae, in addition to F5 and F41, are significantly associated with calf diarrhea, with F5, F17, and F41 isolated 5.7, 1.2, and 23.7 times more frequently from diarrheic calves than from healthy controls, respectively [36,37,38]. F17-carrying ETEC strains are not targeted by any currently licensed maternal vaccine formulation, and their prevalence across geographic regions has not been systematically incorporated into vaccine coverage assessments.

Surveillance of 598 farms affected by neonatal calf diarrhea outbreaks in Northern Italy between 2020 and 2022 detected ETEC in 17.2% of investigated cases, with co-infections involving BRV, BCoV, and *Cryptosporidium* spp. frequently observed and considered to exacerbate clinical severity [39]. Shotgun metagenomic analysis of diarrheic calves from multiple farms in Inner Mongolia identified extensive AMR gene profiles within the intestinal microbiome, including multidrug resistance signatures on farms with no documented history of recent antibiotic use [11]. Whole-genome sequencing of ETEC F5 and F5-F41 strains isolated from neonatal calves in the same region confirmed multidrug resistance to third-generation cephalosporins, fluoroquinolones, and aminoglycosides, agents classified as highest-priority critically important antimicrobials by the World Health Organization, with 74 resistance genes distributed across chromosomes and plasmids in 5 distinct resistance mechanisms [37]. The detection of resistance gene reservoirs independent of documented antibiotic treatment pressure indicates that resistome dissemination in neonatal calf populations may occur through pathways beyond direct therapeutic selection.

The vaccine-relevant implication of ETEC diversity differs structurally from that of BRV and BCoV. Whereas viral antigenic drift generates neutralization-resistant variants against which antibody-based protection fails at the serologic level, ETEC virulence is determined primarily by adhesin expression and enterotoxin gene carriage rather than by surface-protein antigenicity in the classical immunological sense. Current licensed vaccines center on F5/K99 and F41 adhesin antigens, the coverage of which against contemporary field ETEC populations has not been systematically re-evaluated in most major cattle-producing regions since original product registration. Regional surveys documenting adhesin diversity in field isolates support this reframing. In a study of pre-weaned calves in the Republic of Korea, the F17 gene was the most frequently detected ETEC-associated virulence factor, with a prevalence of 72.2%, while F5 and F41 were detected at substantially lower rates [40]. Notably, the authors observed that frequent herd vaccination targeting F5 may itself have contributed to the low F5 prevalence in vaccinated herds, suggesting that selective pressure from F5-targeted vaccines may alter the circulating adhesin profile over time. The vaccine-relevant question for ETEC is therefore not phylogenetic sequence divergence of surface antigens but functional adhesin coverage of circulating strains relative to vaccine antigen composition. This distinction should inform how ETEC surveillance programs are designed and how their outputs are translated into vaccine updating decisions. The practical implication is that adhesin coverage assessments, not phylogenetic analyses of core genome sequences, should constitute the evidentiary standard for ETEC vaccine updating; a standard that does not currently exist in any systematic form for cattle ETEC populations in major markets. A specific question follows from this: at what prevalence of a non-vaccine adhesin such as F17 should vaccine antigen updating be considered? No validated prevalence threshold currently exists, and the evidence indicates that a single prevalence figure would in any case be an insufficient basis for such a decision. Although meta-analysis establishes that F17 is detected significantly more often in diarrheic than in healthy calves [36], F17 is also recovered at high frequency from clinically healthy animals, and in some regional surveys its prevalence in normal feces equals or exceeds that in diarrheic feces [38,41]. High prevalence alone therefore does not establish that an adhesin is driving clinical disease in a given population. A rational updating criterion for ETEC would need to combine adhesin prevalence with the attributable fraction of clinical diarrhea associated with that adhesin, ideally supported by evidence that the adhesin mediates disease rather than asymptomatic colonization. The absence of surveillance designed to generate this combined measure, rather than the absence of a prevalence cutoff alone, is the true gap: no unified standard currently exists for translating ETEC adhesin surveillance into vaccine composition decisions, and establishing one is an unresolved research priority that requires case–control surveillance linking specific adhesins to clinical outcomes, not merely broader prevalence sampling.

The molecular epidemiological evidence reviewed above establishes that all three vaccine-target pathogens are diverging from their respective vaccine reference strains through mechanisms that are biologically distinct for each. Section 4 examines how the passive-transfer cascade imposes additional constraints on field protection, independent of these pathogen-side factors.

## 4. Limitations of Current Maternal Vaccination Programs

### 4.1. The Passive-Transfer Cascade: Sequential Determinants of Field Protection

Prepartum vaccination of pregnant dams is the standard approach to preventing BRV, BCoV, and ETEC in neonatal calves. Vaccination during late gestation stimulates pathogen-specific antibody production in the dam; antibody-enriched colostrum consumed by the calf within hours of birth provides luminal immunoglobulins that reduce enteric pathogen challenge during the period before the calf’s own adaptive immune responses mature. Field and experimental studies have documented increased pathogen-specific immunoglobulin concentrations in colostrum and in calf serum following prepartum vaccination [4,42].

**Figure 4:**
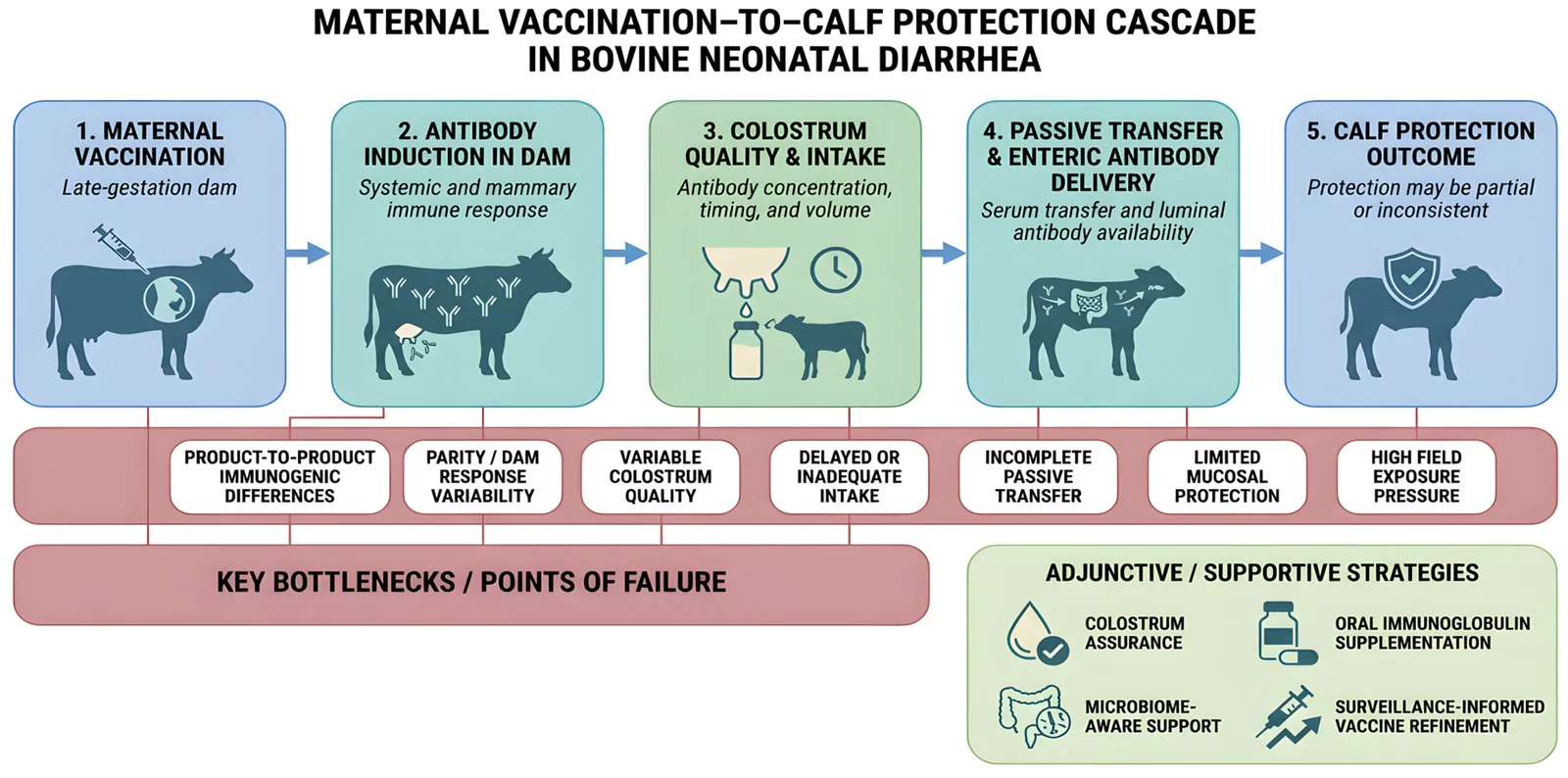
Sequential pathway from maternal vaccination to passive protection in the neonatal calf and principal bottlenecks at each step. Protection in the calf depends on successful progression through five sequential stages: prepartum vaccination of the dam, antibody induction in the mammary gland, colostrum quality and intake volume within the first hours of birth, passive transfer and enteric antibody delivery across the intestinal epithelium before gut closure, and final calf protection outcome. Each stage represents an independently variable factor that can attenuate the protective effect regardless of the immunogenicity of the administered vaccine. Key bottlenecks at each stage are indicated in the lower panel. Adjunctive and supportive strategies that may compensate for specific points of failure are summarized in the lower right.

The protective effect ultimately achieved in the calf, however, depends on events well downstream of vaccination itself. Antibody induction in the dam varies with vaccine formulation, timing of administration relative to parturition, parity, and prior herd pathogen exposure history. The concentration of specific immunoglobulins available to the calf is determined by colostrum yield and immunoglobulin mass per unit volume, the volume consumed at first feeding, and the interval between birth and first feeding. A further constraint is the absorptive capacity of the neonatal intestinal epithelium, which declines linearly from birth and reaches closure at approximately 24 h of age, after which immunoglobulin absorption becomes negligible [43]. Prepartum vaccination has additionally been associated with elevated non-antigen-specific colostral IgM concentrations (8.76 vs. 5.78 mg/mL; *p* = 0.005), suggesting immunostimulatory effects extending beyond targeted pathogen-specific responses, though the clinical significance of this non-specific component remains uncertain [44]. The sequential nature of this protective cascade is illustrated in Figure 4. The clinical relevance of cascade attenuation is reflected in the prevalence of failure of passive transfer under field conditions: a meta-analysis of studies from pasture-based dairy farms in Australasia reported an overall failure of passive transfer (FPT) prevalence of 33% at the individual calf level and 38% at the farm level, indicating that a substantial proportion of calves routinely enter the neonatal challenge period with sub-threshold systemic immunoglobulin concentrations, independent of whether their dams were vaccinated [45]. Comparable rates have been reported in European dairy systems, where 39.7% of 227 calves from 11 Austrian farms met the threshold for FPT, underscoring that passive transfer insufficiency is not geographically restricted [46]. Beyond individual-calf variability, herd-level protection is shaped by population dynamics. Continuous calving, replacement, and animal turnover mean that vaccinated dams, unvaccinated animals, and animals with incompletely established immunity are typically co-housed at any given time. The effective proportion of calves protected within a herd is therefore lower than nominal vaccine coverage figures would suggest, and susceptible animals maintain pathogen circulation that sustains challenge pressure on even well-protected calves. This population heterogeneity operates independently of antigen match and passive-transfer efficiency, and contributes to the persistence of field disease under high nominal vaccination rates. It also provides part of the explanation for field surveillance findings, discussed in Section 3.1, in which pathogen detection rates in vaccinated and unvaccinated herds were comparable [5].

### 4.2. Product-to-Product Variation in Immunogenic Output

Vaccines sharing the same labeled indications do not necessarily produce equivalent immune responses in vaccinated dams or equivalent colostral antibody concentrations available to calves. A comparative study of two commercially available maternal vaccines with overlapping BRV, BCoV, and ETEC coverage found measurable differences in specific antibody concentrations in both colostrum and calf serum, with the most pronounced differences observed for BCoV- and ETEC-specific immunoglobulins [4]. Programs using different commercial products with nominally equivalent pathogen coverage may therefore not be immunologically interchangeable. Field-efficacy comparisons between products are confounded by this source of variation, which operates independently of and before any consideration of field-strain antigen mismatch. A further source of variation operates at the level of the individual dam. Independent of product choice, a proportion of vaccinated dams will not generate a protective antibody response, owing to parity, nutritional status, intercurrent disease, or individual immunological variation. This effect is most pronounced in primiparous animals: in a controlled prepartum vaccination study, calves born to vaccinated dams still developed diarrhea at an incidence of 18.6%, and failure of passive immunity transfer was more frequent in calves born to heifers, which often produce colostrum of lower volume and immunological quality [42]. Even an optimally matched vaccine administered correctly will therefore leave some calves without adequate passively transferred protection, a constraint that no antigen updating strategy can fully overcome.

### 4.3. Mucosal Protection and the Limits of Parenteral Immunization

Parenteral vaccination of the dam generates systemic antibody responses that are subsequently concentrated in colostrum and delivered to the calf’s intestinal lumen after ingestion. This pathway provides luminal IgG at the site of pathogen challenge. Still, it does not reliably induce durable secretory IgA responses in the calf intestinal mucosa, which represents the primary immunological barrier against enteric pathogens at the mucosal surface. Luminal IgG derived from colostrum provides meaningful short-term protection when passive transfer is complete and timely, but this protection is transient, declines as colostrum immunoglobulins are diluted and degraded within the intestinal lumen, and is not supplemented by endogenous mucosal memory responses in the calf. For BCoV specifically, available data suggest that colostral IgG1 antibody titers are associated with protection: calves with serum BCoV-specific IgG1 titers of ≥1024 showed substantially lower rates of infection compared with calves with titers of <1024 in a prospective field study [47]. However, a minimum protective threshold has not been established under controlled challenge conditions, and IgG1 titer alone may not capture the contribution of intestinal IgA to luminal protection [7,8]. Without these thresholds, it is not possible to determine whether a given vaccination program has generated sufficient mucosal immunoglobulin concentrations to protect against field-level pathogen challenge.

This gap has a practical consequence for program evaluation: failure to demonstrate protection in a BCoV field trial cannot be attributed to antigen mismatch rather than insufficient colostral transfer or sub-threshold luminal concentrations without defined protective benchmarks. Until minimum protective titers are established for both serum IgG1 and intestinal IgA, as data suggest both are relevant to enteric BCoV protection, the evidence base for BCoV vaccine evaluation will remain structurally incomplete regardless of the quality of molecular epidemiological data describing circulating strain diversity. Establishing these thresholds through controlled gnotobiotic or colostrum-deprived calf challenge models with titrated antibody transfer is a prerequisite for any rational BCoV vaccine updating process.

### 4.4. The Published Evidence Bases for Licensed Maternal Vaccines

Licensed maternal vaccines against BRV, BCoV, and ETEC retain practical utility when administered according to label directions and integrated with sound colostrum management. A scoping review of 54 publications on vaccination programs for neonatal calf diarrhea in cow-calf operations found the published field-efficacy evidence to be heterogeneous in study design, geographically concentrated in North America and Europe, and inconsistent in outcome measurement, with the majority of publications reporting immunological rather than clinical protection endpoints [6]. Field comparisons between commercial products are further complicated by differences in antigen composition, adjuvant formulation, and administration route across jurisdictions. These limitations do not negate the biological rationale for maternal vaccination, but they preclude confident product-specific efficacy generalizations and underscore the need for standardized clinical endpoints in future vaccine trials.

Against this background, several emerging vaccine platforms and adjunctive strategies have been developed to address specific limitations of conventional maternal vaccination programs.

## 5. Emerging Vaccine Platforms and Adjunctive Strategies

The molecular epidemiological evidence in Section 3 indicates that historically fixed whole-pathogen antigens do not track pathogen populations that continue to diversify through reassortment and epitope-level variation in BRV, receptor-binding domain and spike divergence in BCoV, and adhesin repertoire expansion in ETEC. Antigen selection oriented toward this problem requires characterizing candidate protective antigens for four properties: protective efficacy, epitope diversity, polymorphism across circulating lineages, and cross-protective breadth. This screening framework is summarized by pathogen in Table 2.

The divergence documented for all three pathogens occurs at the level of individual epitopes or adhesins rather than at the level of whole-antigen identity, which is the level at which subunit and multi-epitope antigen design operates. Preclinical constructs of this type have been reported for each vaccine-target pathogen, although none has been evaluated in calves. For BRV, a truncated VP4-derived subunit antigen expressed in *Escherichia coli* was immunogenic in a rat model, following structure-guided in silico design [48]. For BCoV, an in silico study mapped B-cell and T-cell epitopes across the HE, spike, envelope, membrane, and nucleocapsid proteins and assembled them into candidate multi-epitope constructs, without subsequent experimental validation [49]. For ETEC, a multi-epitope fusion antigen incorporating F17, F5, F41, and an STa toxoid epitope was expressed and used to immunize mice; the resulting antiserum inhibited adhesion of F17-, F5-, and F41-positive ETEC strains to intestinal epithelial cells in vitro [50]. These reports establish that antigen content for each pathogen can be defined at the epitope level rather than fixed by whole-virus or whole-cell composition, but the immunogenicity and protective efficacy of such constructs in the target host remain untested.

Unlike fixed whole-pathogen antigens, epitope-defined constructs allow antigen content to be revised as surveillance identifies new circulating variants, which corresponds to the genotype-serology discordance documented for BRV (Section 3.1) and the adhesin-coverage gap documented for ETEC (Section 3.3). Attenuated live vaccines could provide comparably broad and updatable antigen presentation; however, modified-live vaccines are used with caution in pregnant cattle because of the risk of transplacental infection, and the World Organisation for Animal Health advises against their use in pregnant animals. Because BRV, BCoV, and ETEC vaccines are administered to dams in late gestation, this platform would require dedicated reproductive-safety evaluation and is therefore a longer-term rather than a near-term option. Subunit and multi-epitope platforms, together with the VLP and recombinant mucosal approaches discussed in the following subsections, are the more immediate route to updatable antigen composition, subject to the delivery and passive-transfer constraints examined in Section 4 and below.

### 5.1. Virus-like Particle Platforms

Virus-like particle (VLP) technology produces non-replicating antigenic structures that preserve the conformational epitopes of outer-capsid proteins without incorporating a viral genome. For BRV, VP4 and VP7 are the principal targets of neutralizing antibodies, and recombinant VLPs presenting these antigens offer a route to precision antigen design unconstrained by the fixed genotype compositions of conventional inactivated whole-virus vaccines. In a BRV oral challenge model, passive antibodies raised in dams immunized with recombinant VP4/VP7 VLP antigens reduced the incidence of severe diarrhea in challenged calves from 86% to 7% and shortened mean diarrhea duration from 2.7 to 0.9 days [18]. The study was conducted under controlled challenge conditions rather than in naturally exposed field populations, and commercial-scale production and field-efficacy data are not yet available. The findings establish that VLP-derived passive antibodies can reduce clinical disease and that antigen design independent of historical genotype convention is biologically feasible. VLP platforms decouple antigen selection from the constraints of whole-virus cultivation and allow contemporary field strains to be incorporated into the antigen composition, a capability that existing inactivated vaccines structurally lack.

This advantage is confined to antigen design. In the study cited above, the VLP antigens were administered to dams and protection was delivered to calves through colostral passive transfer, so that a VLP-based maternal vaccine would remain subject to the same passive-transfer-cascade limitations described in Section 4: variable colostral antibody concentration, incomplete or delayed passive transfer, and the absence of calf-side mucosal immunity. Improved antigen matching addresses only the pathogen-side component of the vaccine-field strain gap, and when delivered by the maternal route, VLP platforms do not resolve the passive-transfer and mucosal constraints that operate after antigen composition. VLP technology improves the precision of antigen selection, whereas the mucosal and oral approaches considered next address the delivery and mucosal-immunity limitations that antigen design alone cannot overcome.

The economic dimension is also unresolved. Only a small number of VLP-based vaccines have reached commercialization, and large-scale production requires optimized upstream expression, multi-step downstream purification, and stage-by-stage quality control that are more demanding than the processes used for conventional inactivated whole-virus vaccines [51]. No cost or field-scale manufacturing data for bovine enteric VLP vaccines have been published, and whether such products can be manufactured at a price point compatible with routine use in cattle production remains undetermined.

### 5.2. Recombinant Mucosal Delivery Systems

A structural constraint of current maternal vaccination is that the antigen is delivered parenterally to the dam and reaches the calf’s intestinal mucosa only indirectly, through colostral immunoglobulins consumed after birth. This pathway does not engage the gut-associated lymphoid tissue of the calf directly and does not generate calf-side secretory IgA responses at the intestinal epithelial surface. Recombinant mucosal delivery platforms are designed to address this constraint by presenting the antigen directly to the neonatal intestinal immune compartment. A recombinant *Lactobacillus casei* strain engineered to express BRV outer-capsid proteins VP4 and VP7 has been developed as an oral delivery vehicle; protein expression at the expected molecular weights was confirmed by Western blot [19]. In vivo immunogenicity and protective efficacy in calves have not been assessed, and the platform remains at the preclinical stage. The biological rationale is that oral antigen delivery to the calf would bypass the passive-transfer cascade entirely and could induce intestinal secretory IgA responses that parenteral dam vaccination cannot provide.

This advantage applies specifically to direct oral administration to the calf. A recombinant platform administered instead to the dam would deliver protection through colostrum and would therefore remain subject to the same passive-transfer-cascade limitations as conventional and VLP-based maternal vaccines; the mucosal benefit described here depends on the route of administration, not on the recombinant technology itself. The practical barriers to oral calf-delivered platforms are also substantial. Recombinant lactic acid bacteria are genetically modified organisms and are treated cautiously by regulatory agencies despite the established safety of the wild-type strains, so that consistent gut colonization and antigen expression, stability of the live organism during storage and administration, and biological containment to prevent environmental release all remain to be addressed [52]. No field-scale production or cost data are available for such a product in cattle. The biological rationale for oral mucosal delivery is sound, but its economic and field-deployment feasibility is presently unestablished.

### 5.3. Standardized Oral Immunoglobulin Products

Postnatal oral administration of pathogen-specific immunoglobulins addresses the passive-transfer gap directly by supplementing or replacing colostral antibodies at the intestinal lumen without dependence on dam vaccination response, colostrum yield, or the timing of the first feeding. In a randomized controlled field trial involving 489 neonatal calves from 35 herds, oral administration of bovine concentrated lactoserum standardized for antibodies to BRV, BCoV, and ETEC F5 reduced the odds of diarrhea by approximately 67% relative to placebo (OR 0.326; *p* < 0.001), with no deaths in the treatment group compared with four in the placebo group [17]. This constitutes the highest level of clinical evidence currently available for any adjunctive strategy in BND prevention. Oral immunoglobulin products do not replace maternal vaccination, as they address only the passive-transfer step and provide no immunological priming of the dam. Their practical value is greatest when colostral transfer is uncertain, when maternal vaccination history is incomplete, or when pathogen challenge pressure in the calving environment is high.

### 5.4. Adjunctive Strategies Addressing Gaps That Vaccine Updating Alone Cannot Resolve

Even if vaccine antigen compositions were updated to reflect contemporary field strains, passive transfer made complete and consistent, and mucosal correlates of protection fully defined, a residual category of protection failures would remain attributable to host-side factors that vaccination cannot address. These include intestinal dysbiosis that amplifies pathogen susceptibility, AMR in ETEC populations that limits treatment when prevention fails, and inadequate supportive care in early-onset disease. The strategies discussed in this subsection do not substitute for the vaccine improvements identified elsewhere in this review; they address the protection gap that those improvements, even if achieved, would leave. Fluid therapy and correction of electrolyte and acid-base disturbances remain the primary determinants of survival regardless of etiologic agent, and antimicrobial use should be guided by clinical severity and pathogen-specific susceptibility data rather than administered routinely, given the expanding AMR burden documented in ETEC-associated neonatal disease [9,11].

Fecal microbiome profiles of diarrheic calves are characterized by enrichment of Proteobacteria and Enterobacteriales alongside depletion of Firmicutes-associated taxa relative to healthy cohorts [10,20,53]. Whether this dysbiosis precedes and predisposes to enteric disease or develops as a consequence of it has not been resolved, and the answer has direct implications for whether microbiome-targeted interventions are most appropriately positioned as preventives or therapeutics. Administration of *Limosilactobacillus reuteri* strains isolated from healthy calves improved fecal consistency and restored aspects of normal microbiome composition in diarrheic calves [21]. *Limosilactobacillus fermentum* supplementation partially reversed BRV-associated dysbiosis in experimentally affected calves [20]. A compound probiotic preparation reduced *E. coli*-induced diarrhea severity through alterations in tryptophan metabolism and bile acid profiles, with effects that were reproducible in fecal microbiota transplantation experiments in the same model [21,22]. None of these studies constitutes a controlled field trial under natural exposure conditions, and standardization of strain selection, dosing, and clinical endpoints is needed before field-level recommendations can be made.

Gut microbiota-derived ursodeoxycholic acid (UDCA) has been associated with improved intestinal homeostasis in neonatal calves with extended-spectrum beta-lactamase-producing *E. coli* infection, and oral ursodiol attenuated colitis and modulated the TGR5-NF-κB signaling axis in murine models [9]. The mechanistic evidence for UDCA is strongest in murine experiments; calf-specific interventional data remain limited and direct extrapolation to bovine clinical practice is premature. Bacteriophage-based approaches targeting enteric ETEC have been proposed as AMR-compatible alternatives to antibiotic treatment. No controlled efficacy data from naturally occurring calf diarrhea cases have been published, and phage therapy in this context should be regarded as an experimental direction without clinical evidence base at this time.

## 6. Knowledge Gaps and Research Priorities

For BRV, the gap between what current surveillance provides and what vaccine updating requires is the absence of systematic cross-neutralization data against contemporary field isolates. This gap is not a knowledge deficit, as the methodology for cross-neutralization testing is well established, but a structural deficit in how surveillance data are collected and applied. Genotype data are generated routinely because RT-PCR is scalable and inexpensive. Cross-neutralization panels require contemporary field isolates, reference antisera, and cell culture capacity, none of which are currently incorporated into routine BRV surveillance programs in most markets. Published molecular surveillance studies document the genotype composition of circulating strains in detail, but genotype designation does not predict antigenic cross-reactivity, as demonstrated directly by the failure of trivalent vaccine antiserum to neutralize a G6P[5] field strain sharing its VP7 designation with a vaccine component [12]. Serotype surveillance using serum neutralization assays against geographically current isolate panels has not been conducted at a scale that would support evidence-based antigen updating in most major markets. Field detection rate data consistent with limited vaccine coverage [5] provide epidemiological context for this gap but cannot substitute for functional cross-neutralization evidence. Prospective multicenter field-efficacy trials with pre-specified clinical and serological endpoints are also scarce relative to the global burden of BRV-associated disease.

For BCoV, the primary gap is the absence of defined correlates of intestinal protection. Neither minimum protective IgG1 concentrations in the intestinal lumen nor serum neutralization titer thresholds associated with resistance to enteric BCoV challenge have been established quantitatively [7,8]. Without these benchmarks, it is not possible to determine whether a given vaccination program has generated colostral antibody responses sufficient for field-level enteric protection, or whether observed protection failures reflect antigen mismatch, insufficient passive transfer, or sub-threshold mucosal concentrations. Two types of studies are required and are technically feasible with existing methodology. First, correlate-defining studies: gnotobiotic or colostrum-deprived calf challenge experiments that systematically vary the concentration and isotype of passively transferred antibodies and measure viral shedding and clinical outcomes to establish minimum protective thresholds. Second, bridging studies: comparative challenge experiments using both vaccine reference strains and contemporary HE-variant and lineage-divergent field isolates in calves from vaccinated dams, with colostral antibody concentrations measured and controlled, to determine whether documented genetic divergence translates into reduced clinical protection. Neither study type requires methodology beyond what is currently available; both have been absent from the published literature despite decades of commercial BCoV vaccination practice.

For ETEC, the principal unresolved question is whether current vaccine antigen compositions, which center on F5 and F41, provide functional coverage of contemporary field ETEC populations. Regional surveillance data document ETEC strains carrying adhesin types beyond F5 and F41 [38,40], and AMR gene profiles in neonatal calf populations have been documented in the absence of direct treatment pressure [11], indicating that the pathogen landscape relevant to both prevention and treatment has shifted since original vaccine formulations were registered. Surveillance programs combining adhesin typing, enterotoxin gene profiling, and AMR phenotyping across multiple regions are required to quantify the coverage gap and to identify adhesin antigens that should be prioritized in updated formulations.

Across all three pathogens, the gaps identified here map onto the three independently variable factors introduced in Section 1. Pathogen-side divergence requires functional surveillance that generates cross-neutralization data, not merely phylogenetic sequence classification. Passive-transfer-cascade variability requires standardized colostral transfer monitoring as a mandatory component of vaccine field trials, not a background variable. The absence of functional surveillance data capable of guiding antigen updating requires prospective multicenter trials with pre-specified serological and clinical endpoints. Addressing any one of these without the others will produce incremental rather than durable improvements in field protection. The principal evidence gaps and near-term research priorities for each pathogen are summarized in Table 3. 

While the research priorities identified here are necessarily directed at future study design, several actions within current capabilities are warranted immediately. For BRV, surveillance programs in major cattle-producing regions should incorporate cross-neutralization testing against representative contemporary field isolates alongside routine RT-PCR genotyping, using the methodology already demonstrated [12]. For BCoV, field trials of maternal vaccines should pre-specify passive transfer efficiency as a measured covariate rather than treating it as a background condition, enabling retrospective attribution of protection failures. For ETEC, adhesin typing beyond F5 and F41 should be incorporated into outbreak investigation panels, with regional prevalence data compiled as the evidence base for future vaccine composition review. None of these steps requires new technology. Each requires a deliberate reorientation of how surveillance data are collected and what questions field trials are designed to answer.

Translating these surveillance outputs into licensed antigen updates, however, requires a regulatory pathway proportionate to the nature and extent of the antigen change. Experience with antigenically variable veterinary viruses has shown that conventional authorization systems are not always well adapted to vaccines whose antigen composition must track epidemiological need, leading to the multi-strain dossier concept, under which multiple pre-evaluated strains could be maintained within a marketing authorization and incorporated according to current disease risk [54]. Recent veterinary vaccine registration initiatives similarly emphasize that vaccine composition should reflect locally circulating strains, while requiring manufacturers to provide complete dossiers demonstrating product quality, safety, and efficacy [55]. These examples illustrate that surveillance can establish the scientific rationale for antigen replacement but cannot by itself demonstrate that an antigen-modified product retains its approved performance characteristics.

In the absence of a predefined pathogen-specific mechanism, the supporting evidence must address product quality, safety, and efficacy, with the efficacy assessment selected according to the intended product claim and the availability of a validated correlate of protection [56,57]. For maternal enteric vaccines, a scientifically credible data package would therefore need to connect antigenic relevance to functional protection through manufacturing and potency comparability, cross-neutralization or comparative immunogenicity, colostral antibody transfer, and, where necessary, target-animal challenge or field-efficacy evidence. The practical importance of such evidence is illustrated by an analysis of 100 veterinary vaccine applications evaluated through the European Union centralised procedure, in which field efficacy studies had an important or substantial influence on efficacy claims or benefit-risk evaluation in 52 cases [58]. This regulatory constraint does not invalidate surveillance-guided antigen updating but defines the evidence needed to make it operational. A realistic near-term priority is therefore not only to generate the functional surveillance data described above but also to establish, in dialogue with regulatory authorities, a defined and proportionate pathway for enteric vaccine antigen updates, with pre-agreed criteria for antigenic comparability, passive-transfer performance, and clinical protection.

## 7. Conclusions

Bovine neonatal diarrhea persists in vaccinated herds because of three factors operating together: antigenic divergence between vaccine and field strains, variability in the passive-transfer cascade, and the absence of functional surveillance data capable of distinguishing between them. For BRV, cross-neutralization data show that antigenic coverage cannot be inferred from genotype designation, and field detection rates in vaccinated and unvaccinated herds are comparable. For BCoV, field strains have diverged from historical vaccine isolates in the hemagglutinin-esterase receptor-binding domain and spike protein across multiple regions, but the consequence for vaccine efficacy has not been tested under controlled conditions. For ETEC, expanding antimicrobial resistance has altered case management in parallel with the prevention problem, so that stewardship-compatible adjunctive approaches are now part of clinical management.

Maternal vaccination retains biological value and should remain a core component of BND control. The limitations described here are not inherent to the approach; they follow from antigen compositions fixed at registration, pathogen populations that continue to evolve, and the variability of passive-transfer-dependent protection. The protection provided is partial rather than absolute, and partial protection remains clinically and economically valuable even where it does not prevent infection. Eradication of BRV, BCoV, or ETEC through vaccination is unlikely, because these pathogens circulate with environmental and interspecies reservoirs, and continuous calving and co-housing of susceptible animals sustain transmission under high vaccination rates. The objective is therefore improvement in the consistency and adequacy of the partial protection already achieved, rather than eradication.

One component of this improvement is the choice of antigen platform. The molecular divergence documented in Section 3 occurs below the level of whole-pathogen identity: at individual neutralization epitopes for BRV, at localized receptor-binding and spike domains for BCoV, and at individual fimbrial adhesins for ETEC. Subunit and multi-epitope antigen design operates at this same level, which makes these platforms more suited to the observed divergence than inactivated whole-pathogen formulations, whose antigen content cannot readily be revised. Prioritizing these platforms requires, for each pathogen, that candidate protective antigens first be characterized for efficacy, for diversity and polymorphism across circulating lineages, and for cross-protective breadth; this screening framework is summarized in Table 2. Attenuated live vaccines could provide comparably broad and updatable antigen presentation, but modified-live vaccines are used with caution in pregnant cattle because of the risk of transplacental infection, and their use in maternal BND vaccination would require dedicated reproductive-safety evaluation. They are therefore a longer-term rather than a near-term option.

This platform priority applies only to the pathogen-side component of the vaccine–field strain gap. An improved antigen delivered by the maternal route remains subject to the passive-transfer-cascade and mucosal-immunity constraints described in Section 4. Better antigen matching does not resolve variable colostral transfer or the absence of calf-side secretory immunity. Subunit and multi-epitope platforms address the antigen-composition problem; they do not address the other two factors.

Three requirements follow from this review. First, surveillance should generate functional antigenic data rather than sequence classification alone: cross-neutralization for BRV, virus neutralization against lineage-divergent isolates for BCoV, and adhesin surveillance combined with attributable-fraction estimates for ETEC. Second, vaccine field trials should include passive-transfer efficiency as a pre-specified endpoint rather than an uncontrolled variable. Third, adjunctive strategies should be integrated where passive transfer is insufficient. Prioritizing subunit and multi-epitope platforms addresses the first requirement at the level of antigen design; it does not replace the other two.

## Figures and Tables

**Table 1:** Representative Preventive Platforms for Bovine Neonatal Diarrhea and Review-Relevant Limitations.

Platform	Target Pathogen Scope	Deployment Stage	Key Quantitative Evidence	Evidence Level
Licensed maternal vaccines (BRV, BCoV, ETEC)	BRV + BCoV + ETEC F5/K99, F41	Prepartum dam vaccination; commercially available	Immunogenic value documented; field-efficacy evidence heterogeneous across products and regions [4,6]	Observational field studies; scoping review
Standardized oral immunoglobulin products	BRV, BCoV, ETEC F5/K99	Postnatal supplementation; commercially available	OR 0.326 (*p* < 0.001) for diarrhea prevention across 489 calves, 35 herds [17]	Randomized controlled field trial
VLP-based platforms	BRV VP4/VP7	Preclinical/experimental	Diarrhea incidence reduced from 86% to 7% in the challenge model [18]	Experimental challenge model
Recombinant mucosal delivery (*L. casei*)	BRV VP4/VP7	Preclinical	Protein expression confirmed by Western blot; no in vivo efficacy data [19]	Preclinical (in vitro)
Probiotic supplementation	Microbiome stabilization	Experimental/commercial	Improved fecal scores and partial dysbiosis reversal documented [20,21,22]	Experimental; inconsistent across products

Abbreviations: BRV, bovine rotavirus; BCoV, bovine coronavirus; ETEC, enterotoxigenic *Escherichia coli*; VLP, virus-like particle; OR, odds ratio; F5/K99 and F41, fimbrial adhesin antigens targeted by currently licensed vaccines. Evidence levels are classified as follows: randomized controlled field trial, prospective controlled studies conducted under natural field exposure conditions; experimental challenge model, controlled studies using defined pathogen inoculation under experimental conditions; observational field studies, non-randomized studies reporting immunological or clinical outcomes under field conditions; preclinical studies limited to in vitro expression or small-animal models without calf efficacy data. Product availability varies by jurisdiction; “commercially available” refers to products licensed in at least one major cattle-producing market as of the search date.

**Table 2:** Recommended protective-antigen screening framework and prioritized platform for vaccine antigen updating.

Pathogen	Primary Protective Antigen Target	Efficacy Screening Required	Diversity/Polymorphism Screening Required	Cross-Protection Screening Required	Prioritized Platform
BRV	VP4, VP7 neutralizing epitopes	Cross-neutralization titers against contemporary field isolates, benchmarked against a defined protective threshold rather than genotype match alone	Epitope-level antigenic mapping of VP4/VP7 across circulating G6/G8/G10 and P[1]/P[5]/P[11] combinations	Serum panels tested against heterologous G/P combinations to define the breadth of cross-neutralizing coverage	Multi-epitope/subunit VP4–VP7 construct (VLP or recombinant subunit) [18,48]
BCoV	Hemagglutinin-esterase (HE) receptor-binding domain, spike (S) protein	Virus neutralization of contemporary HE-variant and lineage-divergent isolates, with an operational immune-escape threshold as demonstrated for related isolates [35]	Structural and antigenic characterization of HE insertion/deletion variants and S-protein lineage divergence across regions	Cross-neutralization testing across European and Asian-American lineages and regionally divergent isolates	Multi-epitope construct spanning HE, S, E, M, and N structural proteins [49]
ETEC	Fimbrial adhesins (F5/K99, F41, F17) and enterotoxins (STa, LT)	In vitro adhesion-inhibition or challenge-model protection attributable to each adhesin individually, not prevalence alone	Regional adhesin prevalence combined with case–control attributable fraction for clinical diarrhea	Demonstration that anti-adhesin serum cross-inhibits ETEC strains expressing different adhesins	Multivalent adhesin multi-epitope fusion antigen (MEFA) incorporating F5, F41, F17, and toxoid STa [50]

Abbreviations: BRV, bovine rotavirus; BCoV, bovine coronavirus; ETEC, enterotoxigenic *Escherichia coli*; VLP, virus-like particle; MEFA, multiepitope fusion antigen; HE, hemagglutinin-esterase; STa, heat-stable enterotoxin; LT, heat-labile enterotoxin; VP4/VP7, rotavirus outer-capsid proteins; F5/K99, F41, F17, ETEC fimbrial adhesins. Attenuated live vaccines could deliver comparably broad and updatable antigen presentation for all three pathogens; however, modified-live vaccines are used with caution in pregnant cattle because of the risk of transplacental infection, and the World Organisation for Animal Health advises against their use in pregnant animals. Because BND vaccines are administered to dams in late gestation, this platform would require dedicated reproductive-safety evaluation and is considered a longer-term rather than a near-term option.

**Table 3:** Evidence gaps and research priorities for BRV, BCoV, and ETEC in the context of maternal vaccination against bovine neonatal diarrhea.

	Bovine Rotavirus (BRV)	Bovine Coronavirus (BCoV)	ETEC
Primary mechanism of field divergence from vaccine strains	Segment reassortment generating novel VP7/VP4 combinations; interspecies gene exchange	HE receptor-binding domain insertions and deletions; spike protein diversification with regional lineage separation	Adhesin repertoire variation beyond F5/K99 and F41; AMR gene acquisition independent of documented treatment pressure
Strongest available evidence for vaccine-field strain gap	Cross-neutralization failure against G6P[5] and G8P[1] field strains (VN titer < 160) despite shared genotype with vaccine components; equivalent BRV detection in vaccinated (42.1%) and unvaccinated herds (42.6%) [5,12]	Genetic divergence from vaccine reference strains established across European and Asian-American lineages; HE receptor-binding domain structural variants documented in China and the United States [14,15,16,33]	ETEC detected in 17.2% of outbreak farms; MDR signatures in calves without documented antibiotic exposure [11,37,39]
Critical missing evidence	Cross-neutralization data against contemporary geographically relevant field isolates; multicenter field-efficacy trials with clinical endpoints	Challenge studies comparing vaccine efficacy against HE-variant and reference strains; defined minimum protective antibody titers for enteric disease	Regional adhesin typing surveys; systematic AMR phenotyping of field isolates relative to vaccine antigen composition
AMR relevance	Not a primary determinant of vaccine performance	Not a primary determinant of vaccine performance	Central; AMR expansion narrows empirical treatment options and complicates case management
Near-term research priority	Paired genotypic and serological surveillance using cross-neutralization assays against current field isolates to guide antigen updating	Controlled challenge studies with contemporary lineage-divergent strains in calves from vaccinated dams	Regional adhesin and resistance profiling to determine whether current vaccine antigen compositions provide adequate functional coverage

Abbreviations: BRV, bovine rotavirus; BCoV, bovine coronavirus; ETEC, enterotoxigenic *Escherichia coli*; VN, virus neutralization; HE, hemagglutinin-esterase; AMR, antimicrobial resistance; MDR, multidrug resistance; F5/K99 and F41, fimbrial adhesin antigens currently targeted by licensed maternal vaccines.

## Data Availability

No new data were created or analyzed in this study. Data sharing is not applicable to this article.

## Supplementary Materials

The following supporting information can be downloaded at: https://www.mdpi.com/article/10.3390/vaccines14080702/s1. Supplementary Material File S1: Detailed methodology for the structured narrative review; Supplementary Material File S2: Figure-rendering specifications and author-provided inputs; Supplementary Table S1: Summary of studies included in the narrative synthesis (n = 46), organized by pathogen focus.

## Abbreviations

The following abbreviations are used in this manuscript:

BND	bovine neonatal diarrhea
BRV	bovine rotavirus
BCoV	bovine coronavirus
ETEC	enterotoxigenic *Escherichia coli*
VLP	virus-like particle
HE	hemagglutinin-esterase
AMR	antimicrobial resistance
MDR	multidrug resistance
ST	heat-stable enterotoxin
LT	heat-labile enterotoxin
OR	odds ratio
VN	virus neutralization
IgG	immunoglobulin G
IgA	immunoglobulin A
IgM	immunoglobulin M
FPT	failure of passive transfer
WGS	whole-genome sequencing
