# Supplementary material for: The Vaccine–Field Strain Gap in Bovine Neonatal Diarrhea: Molecular Epidemiology of Rotavirus, Coronavirus, and Enterotoxigenic Escherichia coli and Priorities for Vaccine Updating
Source: Vaccines (Basel). 2026 Aug 13;14(8):702. doi: 10.3390/vaccines14080702 (PMC13517803; doi:10.3390/vaccines14080702)
Supplement: Supplementary file 1 [file vaccines-14-00702-s001.zip › vaccines-4438655-supplementary.pdf]

Supplementary Materials File S1. Detailed methodology for the structured narrative review

This review was conducted as a structured narrative review to integrate evidence across bovine rotavirus, bovine coronavirus, and enterotoxigenic Escherichia coli, with emphasis on molecular epidemiology, vaccine–field strain concordance, passive-transfer constraints, and adjunctive control strategies. Structured searches were conducted primarily in PubMed (MEDLINE) for articles published from January 2019 to March 2026. Google Scholar and the reference lists of retrieved articles were used as supplementary sources. The complete PubMed search string is provided in Section 2.2 of the main manuscript.

Eligible sources included peer-reviewed primary research articles and reviews reporting molecular epidemiology, vaccine immunogenicity or efficacy, passive-transfer parameters, colostral antibody responses, or adjunctive interventions relevant to bovine neonatal diarrhea. Publications were excluded when BRV, BCoV, or ETEC were not the primary subject, when the pathogen was outside the scope of licensed maternal vaccines, when the publication was not peer reviewed, or when the full text was not available in English.

Records were screened by title and abstract, followed by full-text assessment of potentially relevant articles. Screening was conducted by the lead author and verified by a co-author, with disagreements resolved through discussion. Because the review was designed as a narrative synthesis rather than a systematic review, formal risk-of-bias scoring and quantitative meta-analysis were not performed. Evidence was instead classified by study design and translational relevance. The study-selection process is presented in Figure 2 using a PRISMA-inspired format, and the characteristics of the 46 included sources are summarized in Supplementary Table S1.

Supplementary Materials File S2. Figure-rendering specifications and author-provided inputs

The specifications below were provided to the figure-rendering tool (FigureLabs). They consist solely of layout, labeling, and formatting instructions. All label text, numerical values, categories, and structural relationships were determined by the authors prior to rendering and are drawn from the sources cited in each figure legend or from the analytical framework of this review. References to visual quality denote formatting intent only and do not imply that the tool contributed scientific judgment, data, or interpretation. The specifications have been lightly edited for clarity and to consolidate redundant iterative instructions; the rendered figures are unchanged.

Figure 1. Vaccine–field strain gap: conceptual overview

Render a clean, flat, vector-style scientific schematic. The figure must be precise, symmetrical, and highly legible, with crisp vector-style icons, accurate English text, generous spacing, and correct alignment. Do not allow misspellings, broken words, duplicated punctuation, overlapping text, overlapping icons, or distorted shapes. Every label must be easy to read.

Use a square canvas with a white background outside the main panels. Build a left-to-right, three-column layout with three tall rounded-rectangle panels. Use soft pastel colors and subtle gradients only: pale blue (left), teal (center), pale green (right). Use dark navy or black icons and text. Keep the style flat, minimal, and schematic. Do not use photorealism, 3D rendering, hand-drawn style, cartoon animals, product branding, or decoration.

At the top center, add a large bold uppercase title: "THE VACCINE–FIELD STRAIN GAP IN BOVINE NEONATAL DIARRHEA". Use the en dash exactly in "VACCINE–FIELD". The title must be centered, large, bold, and fully legible.

LEFT PANEL. Panel title in uppercase: "CURRENT CONTROL INPUTS". Three vertically stacked, evenly spaced items, each with a large icon and a short label.

Item 1: icon of an adult cow being vaccinated with a syringe neatly integrated. Label: "MATERNAL VACCINATION".

Item 2: icon representing colostrum-derived passive immunity (udder, colostrum drop, or antibody motif). Label: "COLOSTRUM-DERIVED PASSIVE IMMUNITY".

Item 3: icon of a calf with a supportive-care or health symbol. Label: "SUPPORTIVE CARE".

All three items centered, aligned, evenly spaced.

CENTER PANEL. Panel title in uppercase: "PROTECTION GAP & BOTTLENECKS" (only one ampersand). Divide visually into an upper half (pathogen-side drivers) and a lower half (host/program bottlenecks).

Upper half: three separated pathogen modules arranged horizontally, each in its own circular or rounded area with clear spacing.

Module 1: heading "BRV"; subheading "Bovine rotavirus"; simple virus/reassortment icon; two labels: "REASSORTMENT" and "GENOTYPE–SEROTYPE DISCORDANCE".

Module 2: heading "BCoV"; subheading "Bovine coronavirus"; coronavirus icon with a branching/divergence motif; two labels: "LINEAGE DIVERGENCE" and "HE VARIATION".

Module 3: heading "ETEC"; subheading "Enterotoxigenic E. coli"; bacterial icon with a resistance symbol; two labels: "ADHESIN DIVERSITY" and "ANTIMICROBIAL RESISTANCE".

Lower half: centered subheading "HOST / PROGRAM BOTTLENECKS", then four horizontal white rectangular boxes stacked vertically, evenly spaced, non-overlapping, each with one label: "VARIABLE COLOSTRUM QUALITY"; "INCOMPLETE PASSIVE TRANSFER"; "PRODUCT-TO-PRODUCT IMMUNOGENIC DIFFERENCES"; "LIMITED MUCOSAL PROTECTION".

The transition from the upper to the lower subsection should suggest narrowing or constraint, using a subtle funnel or narrowing connector, kept clean and simple.

RIGHT PANEL. Panel title in uppercase: "SURVEILLANCE-INFORMED FUTURE CONTROL". Four vertically stacked, evenly spaced items, each with an icon and a label. Use simple arrows or connectors from the center panel toward the right panel.

Item 1: syringe with an update/refresh arrow. Label: "VACCINE UPDATING".

Item 2: colostrum drop with a check mark or magnifier. Label: "IMPROVED COLOSTRUM ASSURANCE".

Item 3: capsule or bottle with a gut/immune motif. Label: "ORAL IMMUNOGLOBULIN SUPPLEMENTATION".

Item 4: gut/microbiome icon. Label: "MICROBIOME-AWARE SUPPORT".

TYPOGRAPHY: modern sans-serif. Bold uppercase for the main title and panel titles; bold or semi-bold for module headings (BRV, BCoV, ETEC); slightly smaller but clearly readable type for subheadings and labels. Prioritize text clarity over icon complexity. Maintain generous spacing. Visual hierarchy: main title, panel titles, module headings, labels.
CRITICAL TEXT ACCURACY: render all of the following exactly, with no spelling errors and no broken words: THE VACCINE–FIELD STRAIN GAP IN BOVINE NEONATAL DIARRHEA; CURRENT CONTROL INPUTS; MATERNAL VACCINATION; COLOSTRUM-DERIVED PASSIVE IMMUNITY; SUPPORTIVE CARE; PROTECTION GAP & BOTTLENECKS; BRV; Bovine rotavirus; REASSORTMENT; GENOTYPE–SEROTYPE DISCORDANCE; BCoV; Bovine coronavirus; LINEAGE DIVERGENCE; HE VARIATION; ETEC; Enterotoxigenic *E. coli*; ADHESIN DIVERSITY; ANTIMICROBIAL RESISTANCE; HOST / PROGRAM BOTTLENECKS; VARIABLE COLOSTRUM QUALITY; INCOMPLETE PASSIVE TRANSFER; PRODUCT-TO-PRODUCT IMMUNOGENIC DIFFERENCES; LIMITED MUCOSAL PROTECTION; SURVEILLANCE-INFORMED FUTURE CONTROL; VACCINE UPDATING; IMPROVED COLOSTRUM ASSURANCE; ORAL IMMUNOGLOBULIN SUPPLEMENTATION; MICROBIOME-AWARE SUPPORT.
ERROR PREVENTION: do not misspell BOTTLENECKS, GENOTYPE, SEROTYPE, DISCORDANCE, ANTIMICROBIAL, IMMUNOGENIC, IMMUNOGLOBULIN, MICROBIOME. Do not duplicate punctuation or the ampersand. Do not merge words, overlap text with icons, overlap pathogen modules, or crowd the bottleneck boxes. Do not distort the cow, calf, virus, bacteria, or gut icons.
STYLE GOAL: clean, minimal, balanced, and legible, with a consistent flat schematic style throughout.

Figure 2. Study Selection Flow

Render a study-selection flow diagram in a clean, flat, schematic style. Vertical top-to-bottom flow with three stages. No title text inside the figure.
Layout: a vertical center column of main boxes connected by downward arrows. One exclusion box offset to the right, connected by a horizontal arrow. Three stage labels (IDENTIFICATION, SCREENING, INCLUDED) in a vertical band on the left, aligned with the corresponding rows.
STAGE 1 — IDENTIFICATION
Main box (top center): "Records identified through PubMed search, January 2019 to March 2026 (n = 268)"
Box below (center): "Additional records identified through reference-list screening and foundational pre-2019 sources (included where relevant)"
STAGE 2 — SCREENING
Main box (center): "Records screened by title and abstract (n = 268)"
Exclusion box (offset right, gray): "Records excluded (n = 222): not BRV, BCoV, or ETEC as primary subject; pathogen outside licensed maternal vaccine scope; no relevant molecular, vaccine, or passive-transfer data; not peer-reviewed or not in English" (horizontal arrow from the screening box to this exclusion box)
STAGE 3 — INCLUDED
Main box (bottom center): "Studies included in narrative synthesis (n = 46): BRV (n = 14); BCoV (n = 8); ETEC (n = 5); cross-cutting — passive transfer, colostrum, microbiome, AMR, and methodology (n = 19)"
Style: main boxes light blue fill (#D5E4F0) with dark blue border (#2166AC); exclusion box light gray fill (#EEEEEE) with gray border; left stage-label band darker blue (#2166AC) with white text. Font: sans-serif, minimum 9pt, black text in boxes. White background, downward arrows between main boxes, one horizontal arrow to the exclusion box. No shadows, no 3D effects. Portrait orientation.

Figure 3. Cross-neutralization

Render a two-panel scientific figure, panels side by side. No figure legend, caption, or title text inside the figure. Panel A on the left (about 65% width), Panel B on the right (about 35% width). White background, clean minimal flat style, no outer border lines around the panels.
Panel A — grouped bar chart with error bars, logarithmic Y-axis. Panel label "(A)" at upper left.
Y-axis: "Virus neutralization (VN) titer", log scale, range 100 to 10,000.
X-axis: four field strains, each with strain name on the top line and genotype below: AH1471 (G10P[11]); Shimane (G6P[1]); AH1207 (G6P[5]) with AH1207 and G6P[5] in bold; AH1041 (G8P[1]).
Bars and values: AH1471: range 3,620–8,791, plotted at midpoint with a min–max error bar, solid blue (

#2a78d6).
Shimane: range 3,620–8,791, plotted at midpoint with a min–max error bar, solid blue (
#2a78d6).
AH1207: range 160–199, plotted at midpoint with a min–max error bar, solid red (
#e24b4a).
AH1041: below 160, bar top at approximately 120 (clearly below the threshold line), with a "<" symbol and a downward arrow inside the bar, solid red (
#e24b4a).
Dashed horizontal line at VN titer = 160, labeled "VN titer = 160" to the left of the line only.
A single callout arrow pointing to the AH1207 bar with the text: "Same G-type (G6) as vaccine component — neutralization failed".
Small text box near the top: "Antiserum: G6G10P[1][5][11] trivalent vaccine".
Legend: blue = "Neutralized"; red = "Neutralization failure".
Small source note at the bottom of the panel: "Adapted from Hasan et al., J Vet Med Sci, 2023".
Panel B — simple paired bar chart, linear Y-axis. Panel label "(B)" at upper left.
Y-axis: "BRV detection rate (%)", range 0–60%.
X-axis: "Vaccinated" and "Unvaccinated".
Bars: Vaccinated 42.1% (solid blue
#2a78d6); Unvaccinated 42.6% (solid gray
#888780). Value labels above each bar. A bracket connecting the two bars labeled "ns".
Small source note at the bottom of the panel: "Adapted from Cho et al., Infect Genet Evol, 2022".
Font: sans-serif, minimum 9pt, all text legible. No gridlines except Y-axis reference lines. No shadows or 3D effects.

Figure 4. Maternal vaccination-to-calf protection cascade

Render a flat, vector-style scientific schematic in the same visual style as the conceptual overview figure already produced for this manuscript (white background, rounded rectangular panels, soft pastel blue/teal/green palette, flat vector icons, bold uppercase section titles, generous spacing). Match that figure in line thickness, icon treatment, corner rounding, spacing, arrow style, color softness, and typography weight. Do not use photorealism, hand-drawn style, cartoon styling, product branding, distorted text, overlap, or misspellings.
STRUCTURE: the figure shows a five-stage sequential pathway from dam vaccination to calf protection outcome (upper cascade band), a lower band listing bottleneck points aligned beneath the relevant stages, and a smaller side panel listing adjunctive strategies. The content of each element is specified below.
TITLE: at the top center, a large bold uppercase title: "MATERNAL VACCINATION–TO–CALF PROTECTION CASCADE IN BOVINE NEONATAL DIARRHEA". Use en dashes exactly in "VACCINATION–TO–CALF". Centered, bold, legible.
UPPER CASCADE: five large rounded rectangular modules arranged horizontally left to right, connected by four clean blue arrows (1 → 2 → 3 → 4 → 5), evenly spaced and aligned, in a soft blue-to-teal tone. Each module contains one heading, one short subheading, and one icon. Do not repeat any explanatory text within a module or add duplicate captions.
Stage 1: heading "1. MATERNAL VACCINATION"; subheading "Late-gestation dam"; icon of a maternal/pregnant cow receiving vaccination, syringe integrated near the shoulder, no detached human hand.
Stage 2: heading "2. ANTIBODY INDUCTION IN DAM"; subheading "Systemic and mammary immune response"; icon of a cow silhouette with antibody symbols oriented toward the mammary region.
Stage 3: heading "3. COLOSTRUM QUALITY & INTAKE"; subheading "Antibody concentration, timing, and volume"; icon of an udder/colostrum droplet with an intake cue and a small clock symbol.
Stage 4: heading "4. PASSIVE TRANSFER & ENTERIC ANTIBODY DELIVERY"; subheading "Serum transfer and luminal antibody availability"; icon of a neonatal calf with a simplified intestine and antibody symbols, uncluttered.
Stage 5: heading "5. CALF PROTECTION OUTCOME"; subheading "Protection may be partial or inconsistent"; icon of a calf with a shield/health-protection symbol.

LOWER BAND: a separate lower rounded rectangular band in a muted rose / pale salmon color. Title: "KEY BOTTLENECKS / POINTS OF FAILURE". Below the title, seven rounded white boxes, evenly spaced and clearly separated, each connected upward to the relevant stage with a thin, clean connector line.
Box labels and alignment: "PRODUCT-TO-PRODUCT IMMUNOGENIC DIFFERENCES" (beneath the Stage 1–2 transition); "PARITY / DAM RESPONSE VARIABILITY" (beneath Stage 2); "VARIABLE COLOSTRUM QUALITY" (beneath Stage 3); "DELAYED OR INADEQUATE INTAKE" (beneath the Stage 3–4 transition); "INCOMPLETE PASSIVE TRANSFER" (beneath Stage 4); "LIMITED MUCOSAL PROTECTION" (beneath the Stage 4–5 transition); "HIGH FIELD EXPOSURE PRESSURE" (beneath Stage 5). Keep this band secondary to the main cascade: fully readable but not heavier than the top band.

LOWER RIGHT PANEL: a smaller pale green rounded panel on the lower right, secondary to the cascade. Title: "ADJUNCTIVE / SUPPORTIVE STRATEGIES". Four items in a balanced 2×2 grid or a clean vertical layout, each with a small flat icon and a short label, generous spacing, no overlap.
Labels and icons: "COLOSTRUM ASSURANCE" (droplet with check mark); "ORAL IMMUNOGLOBULIN SUPPLEMENTATION" (capsule or bottle); "MICROBIOME-AWARE SUPPORT" (intestine/microbiome icon); "SURVEILLANCE-INFORMED VACCINE REFINEMENT" (syringe with an update arrow or a simple data/update motif).
TYPOGRAPHY: modern sans-serif. Bold uppercase for the main title, section titles, and stage headings; smaller but clearly readable text for subheadings and labels. Prioritize readability; reduce icon complexity rather than text size.
CRITICAL TEXT ACCURACY: render all of the following exactly, with no spelling errors, broken words, duplicated punctuation, or overlapping lines: MATERNAL VACCINATION–TO–CALF PROTECTION CASCADE IN BOVINE NEONATAL DIARRHEA; 1. MATERNAL VACCINATION; Late-gestation dam; 2. ANTIBODY INDUCTION IN DAM; Systemic and mammary immune response; 3. COLOSTRUM QUALITY & INTAKE; Antibody concentration, timing, and volume; 4. PASSIVE TRANSFER & ENTERIC ANTIBODY DELIVERY; Serum transfer and luminal antibody availability; 5. CALF PROTECTION OUTCOME; Protection may be partial or inconsistent; KEY BOTTLENECKS / POINTS OF FAILURE; PRODUCT-TO-PRODUCT IMMUNOGENIC DIFFERENCES; PARITY / DAM RESPONSE VARIABILITY; VARIABLE COLOSTRUM QUALITY; DELAYED OR INADEQUATE INTAKE; INCOMPLETE PASSIVE TRANSFER; LIMITED MUCOSAL PROTECTION; HIGH FIELD EXPOSURE PRESSURE; ADJUNCTIVE / SUPPORTIVE STRATEGIES; COLOSTRUM ASSURANCE; ORAL IMMUNOGLOBULIN SUPPLEMENTATION; MICROBIOME-AWARE SUPPORT; SURVEILLANCE-INFORMED VACCINE REFINEMENT.
ERROR PREVENTION: do not misspell IMMUNOGENIC, VARIABILITY, COLOSTRUM, INADEQUATE, PASSIVE, MUCOSAL, IMMUNOGLOBULIN, MICROBIOME, SURVEILLANCE, REFINEMENT. Do not repeat text within stage panels, overlap text with icons, crowd the bottleneck boxes, over-densify the adjunctive panel, or distort animal shapes.
FINAL GOAL: a clean, legible schematic matching the style of the conceptual overview figure, clearly showing the five-stage cascade, the bottleneck band, and the adjunctive strategies panel, with all text rendered exactly as specified.

Supplementary Table S1. Summary of studies included in the narrative synthesis (n = 46), organized by pathogen focus.

Ref.	First author (year)	Study design	Region	Pathogen focus	Key finding relevant to the review
**Bovine rotavirus (BRV) — 14 studies**					
[5]	Cho et al. (2022)	Molecular surveillance	Republic of Korea	BRV	G6/G8/G10 co-circulation with reassortment; BRV detection comparable in vaccinated (42.1%) vs unvaccinated (42.6%) herds
[12]	Hasan et al. (2023)	Cross-neutralization assay	Japan	BRV	G6P[5] strain (AH1207) escaped neutralization despite sharing VP7 genotype with vaccine component
[18]	Dhama et al. (2009)	Review	Global scope	BRV	Foundational review of rotavirus diarrhea in bovines; VP7/VP4 define G and P genotypes
[19]	Gichile (2022)	Review	Global scope	BRV	Review of BRV epidemiology and public health significance
[20]	Fritzen et al. (2019)	Cross-sectional genotyping	Brazil	BRV	Vaccinated herds carried G6P[11]/G10P[11], not in the G6P[5] vaccine in use
[21]	Hasan et al. (2022)	Longitudinal genotyping	Japan	BRV	Genotype composition in a vaccinated farm differed from the vaccine formulation (2018–2020)
[22]	Ghonaim et al. (2025))	Whole-genome characterization	China	BRV	G6P[1] strain with segments related to human/feline rotaviruses; interspecies reassortment
[23]	Louge Uriarte et al. (2023)	Molecular epidemiology	South America	BRV	Spatiotemporal diversification of G6 lineages; G8P[11] strain characterized
[24]	Singh et al. (2022)	Genetic characterization	United States	BRV	Novel bovine G37P[52] strain related to human rotaviruses; interspecies gene exchange
[25]	Barua et al. (2024)	Whole-genome constellation	Bangladesh	BRV	Dominant G6P[11] lineage with NSP4 intragenic recombination with human sequences
[26]	Fritzen et al. (2019)	Longitudinal surveillance	Brazil	BRV	Dominant field strain shifted G6-type → G10P[11] after G6P[5] vaccine introduction
[27]	Hoshino & Kapikian (2000)	Review	Global scope	BRV/BCoV	Foundational serotype-genotype account; VP7 variation alters neutralization epitopes without changing genotype
[46]	Bristol et al. (2021)	Experimental challenge model	United States	BRV	VP4/VP7 VLP-derived passive antibodies reduced severe diarrhea 86%→7%; shortened duration
[48]	Atalay et al. (2025)	Preclinical (in vitro)	Türkiye	BRV	Recombinant *L. casei* expressing BRV VP4/VP7 confirmed by Western blot; oral candidate
**Bovine coronavirus (BCoV) — 8 studies**					
[7]	Vlasova & Saif (2021)	Review	Global scope	BCoV	Comprehensive BCoV review; partial genetic overlap between enteric and respiratory strains

[8]	Zhu et al. (2022)	Review	Global scope	BCoV	Review of BCoV epidemiology and lineage diversification
[14]	Abi et al. (2020)	Molecular characterization	China	BCoV	First BCoV variant carrying a 12-nucleotide insertion in the HE receptor-binding domain
[15]	Workman et al. (2022)	Molecular surveillance	United States	BCoV	HE receptor-binding domain insertion/deletion variants detected in US cattle
[16]	Zhao et al. (2025)	Isolation and characterization	China	BCoV	HE-variant strains in dairy calves; molecular docking suggested altered binding affinity for O-acetylated sialic acid
[28]	Kim et al. (2022)	Molecular surveillance	Republic of Korea	BCoV	Korean BCoV strains in the Asia/USA lineage with elevated nucleotide substitution rates
[29]	Yilmaz et al. (2025)	Molecular characterization	Türkiye (comparative)	BCoV	Vaccine-field similarity varied by region: 96.8-100% (Thrace) vs 96.0-97.8% (Korea/US) to the vaccine reference strain
[42]	Bok et al. (2018)	Prospective field study	Argentina	BCoV	Calves with serum BCoV IgG1 titers >=1024 showed lower infection rates; titer associated with protection but no defined threshold
**Enterotoxigenic *Escherichia coli* (ETEC) — 5 studies**					
[31]	Foster & Smith (2009)	Review	United States	ETEC	Foundational review of diarrhea pathophysiology in calves
[32]	Xie et al. (2026)	Whole-genome sequencing	China	ETEC	F5 and F5-F41 strains MDR to 3rd-gen cephalosporins, fluoroquinolones, aminoglycosides; 74 resistance genes
[33]	Kolenda et al. (2015)	Systematic review / meta-analysis	27 countries	ETEC	F5, F17, F41 significantly associated with calf diarrhea; F17 relevant beyond vaccine-targeted F5/F41
[34]	Torreggiani et al. (2025)	Outbreak surveillance	Italy	ETEC	ETEC in 17.2% of 598 outbreak farms; frequent co-infection with BRV, BCoV, Cryptosporidium
[35]	Ryu et al. (2020)	Virulence-gene characterization	Republic. of Korea	ETEC	F17 most frequent adhesin gene (72.2%); F5-targeted vaccination may have lowered F5 prevalence
**Cross-cutting (Passive transfer, Colostrum, Microbiome, AMR, and Methodology) — 19 studies**					
[1]	Urie et al. (2018)	National longitudinal study	United States	Cross-cutting	Digestive disorders accounted for 56.0% of disease events; 5.0% preweaning mortality
[2]	Cho & Yoon (2014)	Review	Global scope	Cross-cutting	Overview of calf diarrhea etiology, diagnosis, and intervention
[3]	Brunauer et al. (2021)	Meta-analysis	21 countries	Cross-cutting	Co-infection: BRV-BCoV 2.84%, BRV-ETEC 1.64%, BRV-Cryptosporidium 6.69%
[6]	Maier et al. (2022)	Scoping review	North America / Europe	Cross-cutting	Field-efficacy evidence heterogeneous, mostly immunological endpoints
[9]	He et al. (2022)	Experimental (murine + calf)	China	Cross-cutting	Gut microbiota-derived UDCA improved homeostasis; ursodiol modulated TGR5–NF-κB axis

[10]	Jessop et al. (2024)	Review	Global scope	Cross-cutting	Review of calf diarrhea and gut microbiota manipulation
[11]	Li et al. (2025)	Metagenomic analysis	China	Cross-cutting	Extensive AMR gene profiles incl. MDR without recent antibiotic use
[13]	de Rooij et al. (2025)	Comparative analysis	US / Europe / Oceania	Cross-cutting	Influenza strain selection uses HI-based antigenic characterization; surveillance precedent
[17]	Nussbaum et al. (2023)	Randomized controlled field trial	Europe	Cross-cutting	Oral standardized Ig reduced diarrhea odds ~67% (OR 0.326; P<0.001), 489 calves, 35 herds
[4]	Gonzalez et al. (2021)	Comparative field study	Spain	Cross-cutting	Two maternal vaccines differed in colostral/serum antibody, most for BCoV and ETEC
[37]	Pinheiro et al. (2022)	Controlled prepartum vaccination study	Brazil	Cross-cutting	Calves from vaccinated dams still developed diarrhea (18.6%); FPT more frequent in heifers' calves
[38]	Godden et al. (2019)	Review	North America	Cross-cutting	Intestinal Ig absorption declines to closure at ~24 h of age
[39]	Chambers et al. (2022)	Field study	New Zealand	Cross-cutting	Prepartum vaccination raised non-specific colostral IgM (8.76 vs 5.78 mg/mL; *P=0.005*)
[40]	Van et al. (2023)	Meta-analysis	Australasia	Cross-cutting	FPT prevalence 33% (calf level), 38% (farm level)
[41]	Hartsleben et al. (2023)	Diagnostic evaluation study	Austria	Cross-cutting	FPT in 39.7% of 227 calves across 11 farms
[50]	Murtaza et al. (2024)	Experimental (16S metagenomics)	Pakistan	Cross-cutting	*L. fermentum* partially reversed BRV-associated dysbiosis
[51]	Ku et al. (2025)	Microbiome profiling	Republic of Korea	Cross-cutting	Gut microbiome alterations in diarrheic Korean calves; dysbiosis may persist
[52]	Zhai et al. (2024)	Experimental (ML-enhanced)	United States	Cross-cutting	*L. reuteri* from healthy calves improved fecal consistency and microbiome
[53]	Zhong et al. (2026)	Experimental + FMT	China	Cross-cutting	Compound probiotics reduced E. coli diarrhea via tryptophan/bile acid metabolism

Abbreviations: BRV, bovine rotavirus; BCoV, bovine coronavirus; ETEC, enterotoxigenic *Escherichia coli*; VLP, virus-like particle; HE, hemagglutinin-esterase; AMR, antimicrobial resistance; MDR, multidrug resistance; FPT, failure of passive transfer; FMT, fecal microbiota transplantation; UDCA, ursodeoxycholic acid; Ig, immunoglobulin; HI, hemagglutination inhibition; ML, machine learning. Reference numbers correspond to the reference list of the main manuscript.

Note: The 46 studies listed above were identified through the structured search and screening process (Section 2, Figure 2). Three additional proof-of-concept antigen-design references discussed in Section 5, identified through reference-list screening rather than the primary database search, are not included in this count: a VP4-derived subunit antigen for BRV [43], an in silico multi-epitope design for BCoV [44], and a multi-epitope fusion antigen for ETEC [45].
